# Molecular characterization of pyridoxine 5′-phosphate oxidase and its pathogenic forms associated with neonatal epileptic encephalopathy

**DOI:** 10.1038/s41598-020-70598-7

**Published:** 2020-08-12

**Authors:** Anna Barile, Isabel Nogués, Martino L. di Salvo, Victoria Bunik, Roberto Contestabile, Angela Tramonti

**Affiliations:** 1 Istituto di Biologia e Patologia Molecolari, Consiglio Nazionale delle Ricerche, Rome, Italy; 2 Dipartimento di Scienze Biochimiche “A. Rossi Fanelli”, Sapienza Università di Roma, Rome, Italy; 3 Istituto di Ricerca sugli Ecosistemi Terrestri, Consiglio Nazionale delle Ricerche, 00015 Monterotondo, Rome, Italy; 4 Belozersky Institute of Physico-Chemical Biology, Faculty of Bioengineering and Bioinformatics, Lomonosov Moscow State University, Moscow, 119991 Russia; 5 Department of Biochemistry, Sechenov University, Trubetskaya, 8/2, Moscow, 119991 Russia

**Keywords:** Enzyme mechanisms, Enzymes, Biochemistry, Diseases, Neurological disorders

## Abstract

Defects of vitamin B_6_ metabolism are responsible for severe neurological disorders, such as pyridoxamine 5′-phosphate oxidase deficiency (PNPOD; OMIM: 610090), an autosomal recessive inborn error of metabolism that usually manifests with neonatal-onset severe seizures and subsequent encephalopathy. At present, 27 pathogenic mutations of the gene encoding human PNPO are known, 13 of which are homozygous missense mutations; however, only 3 of them have been characterised with respect to the molecular and functional properties of the variant enzyme forms. Moreover, studies on wild type and variant human PNPOs have so far largely ignored the regulation properties of this enzyme. Here, we present a detailed characterisation of the inhibition mechanism of PNPO by pyridoxal 5′-phosphate (PLP), the reaction product of the enzyme. Our study reveals that human PNPO has an allosteric PLP binding site that plays a crucial role in the enzyme regulation and therefore in the regulation of vitamin B_6_ metabolism in humans. Furthermore, we have produced, recombinantly expressed and characterised several PNPO pathogenic variants responsible for PNPOD (G118R, R141C, R225H, R116Q/R225H, and X262Q). Such replacements mainly affect the catalytic activity of PNPO and binding of the enzyme substrate and FMN cofactor, leaving the allosteric properties unaltered.

## Introduction

The active form of vitamin B_6_, pyridoxal 5′-phosphate (PLP), acts as cofactor for about 150 different enzymes found across all species, representing 4% of all known catalytic activities^1^. Among these, in humans several are involved in the biosynthesis and degradation of amino acids, biogenic amines, and neurotransmitters in the brain, including dopamine, γ-aminobutyric acid (GABA), serotonin, histamine, d-serine, and epinephrine^2^. Mammalian cells are not able to carry out the de novo synthesis of PLP, but recycle it from the other B_6_ vitamers—such as pyridoxine (PN), pyridoxamine (PM), and pyridoxal (PL)—supplied in the diet, or deriving from degraded enzymes, in a so-called salvage pathway. A key step in the formation of PLP—the oxidation of either pyridoxine 5′-phosphate (PNP) or pyridoxamine 5′-phosphate (PMP)—is catalysed by pyridoxine 5′-phosphate oxidase (PNPO, EC 1.4.3.5; accepted name pyridoxal 5′-phosphate synthase), an FMN-dependent enzyme, using molecular oxygen as the final electron acceptor. The PLP salvage pathway also comprises an enzyme called pyridoxal kinase (PLK), which phosphorylates PN, PM and PL, and several phosphatases which catalyse the dephosphorylation of PLP, PNP and PMP^3^.

PNPO catalyses the direct transfer of a pair of electrons, in the form of hydride ion, from the C4′ of PNP or PMP to a tightly bound molecule of FMN, forming FMNH_2_^4^. These electrons are then transferred in a second half-reaction to molecular oxygen, regenerating FMN and forming H_2_O_2_. The human enzyme is a homodimer and the corresponding gene is located on chromosome 17q21.2^5^. Characterization of recombinant human PNPO showed that this enzyme, similarly to *Escherichia coli* PNPO, contains a secondary, non-catalytic site that tightly binds PLP^6^. We have recently demonstrated that *E. coli* PNPO is inhibited by PLP when this binds at an allosteric site, whose location on the enzyme structure is unknown and that may correspond to the secondary site indicated by other authors^7^. Analogously, it has been reported that human PNPO, which is structurally very similar to its *E. coli* homologue, is inhibited by PLP, although this inhibition has been attributed to PLP binding at the active site^6^. In this work, we carried out a kinetic characterization of human PNPO with the aim to check if the allosteric inhibition observed with the *E. coli* enzyme might also apply to the human orthologue. This feature is of particular importance in the regulation of human vitamin B_6_ metabolism, also because the ability to retain PLP in a tight binding site may be related to the transfer mechanism of this vitamer to the apo-forms of vitamin B_6_-dependent enzymes^8^.

Maintenance of a correct balance among B_6_ vitamers inside the cell is of fundamental physiological importance in all organisms, including humans where PLP imbalance leads to severe neurological dysfunctions^9^. Among these, of particular significance is pyridoxamine 5′-phosphate oxidase deficiency (PNPOD; OMIM: 610090), a severe disorder which usually manifests a few hours after birth with seizures that do not respond to conventional anticonvulsant treatments. This particular form of epilepsy is caused by mutations in human *PNPO*, that lead to an imbalance of PLP homeostasis and a consequent decreased activity of PLP-dependent enzymes, as shown by the analysis of metabolites present in the cerebrospinal fluid and urine of affected patients^10^. PNPOD shows symptoms such as fetal distress, hypoglycinemia, anemia, acidosis, and asphyxia, that can be successfully treated with PLP and, in some cases, with PN^11^.

To date, 27 pathogenic mutations of the gene encoding human PNPO have been genetically confirmed in 62 patients^11^. Among these, 13 are homozygous missense mutations (Supplementary Table 1), but also stop codon suppression, nonsense changes introducing a premature stop codon, deletions, and splice site mutations have been reported in the literature^10–18^. So far, the only PNPOD-related human PNPO variants characterized from a functional and structural point of view are R229W^15^, R95C^8^ and R116Q^19^. In particular, R95C and R229W variants are much less catalytically efficient than the wild type enzyme, and also presented a large reduction in affinity for the FMN cofactor. The crystal structure of the R229W variant showed that the replacement prevents the proper binding of both PNP substrate and FMN cofactor^15^. Recently, the functional effects of the c.347G>A (p.R116Q) mutation of human *PNPO* gene have been studied. The peculiarity of this mutation is that in most cases patients present a late epilepsy onset—beyond the neonatal period. The R116Q variant is endowed with a 40% residual catalytic efficiency, has a 20-fold reduced affinity for FMN, and is more susceptible to thermal denaturation; all these features could account for the relatively mild epilepsy phenotype associated with this variant^19,20^.

**Table 1:** Parameters obtained from kinetic and equilibrium measurements on PNPO forms.

PNPO enzyme form	FMN binding		K_M_ (μM)	k_CAT_ (s^−1^)	PNPO activity	PLP inhibition	
	Saturation (%)	K_D_ (nM)				K_I_ or K_D1_ (μM)	εK_I_ or K_D2_ (μM)
Wild type (TRIS)	77 ± 3	13.1 ± 1.7^1^	2.6 ± 0.2	0.062 ± 0.002	100%	0.62 ± 0.09^a^	4.07^a^
Wild type (HEPES)			2.8 ± 0.2	0.057 ± 0.002		0.95 ± 0.01^b^	3.2 ± 0.2^c^
G118R	44 ± 6	94 ± 19	5.7 ± 0.8^b^	0.022 ± 0.001^b^		0.26 ± 0.03^b^	n.d
R141C	57 ± 10	1,310 ± 260	6.1 ± 0.8^b^	0.019 ± 0.001^b^	51%^2^	0.96 ± 0.05^b^	1.5 ± 0.3^c^
R225H	76 ± 6	n.d	16 ± 2^b^	0.002 ± 7 × 10^−5b^	0%^2^ 8%^3^	0.87 ± 0.17^b^	14.5 ± 1.1^c^
R116Q/R225H	76 ± 8	n.d	5.1 ± 0.7^b^	0.012 ± 3 × 10^−4b^		0.71 ± 0.04^b^	6.4 ± 0.9^c^
X262Q	22 ± 6	2,400 ± 420	n.d	0	0%^4^ 12%^3^	0.94 ± 0.05^b^	n.d
R95C		354 ± 12^5^	436 ± 35^5^	0.037 ± 0.001^5^			
R116Q		251 ± 41^1^	3.1 ± 0.2^1^	0.037 ± 5 × 10^−4 1^	83%^3^		
R229W		672 ± 65^6^	461 ± 27^6^	0.040 ± 0.001^6^	30%^4^ 15%^3^		

*n.d.* not detectable.

^a^K_I_ determined from PLP inhibition kinetic studies.

^b^K_D1_ determined from PLP binding fluorimetric analysis.

^c^K_D2_ determined by DSF assay.

^1^From^19^.

^2^From^21^ PNPO activity was measured in CHO-K1 cell lysates using PMP as substrate.

^3^From^12^ PNPO activity was measured in HeLa cell lysate systems using PMP as substrate.

^4^From^13^ PNPO activity was measured in CHO cell lysates using PMP as substrate.

^5^From^8^.

^6^From^15^.

In this work we have characterized at a molecular level other missense mutations found in children affected by PNPOD (Supplementary Table 1). In particular, the PNPO G118R, R141C, R225H, R116Q/R225H, and X262Q variants were recombinantly expressed, purified and characterized in order to precisely understand how these replacements affect protein stability and catalytic activity, but also whether they perturb the affinity for FMN and PLP inhibition properties. A detailed functional characterization of these variants is very important for a full understanding of the molecular bases of the disease and for the rational design of treatment strategies. In particular, it is necessary to understand the reasons behind the opportunity to treat patients with PN, PLP or vitamin B_2_ (riboflavin).

## Results

### Identified mutations in human PNPO show different clinical outcomes

All PNPO variants found in patients affected with PNPOD reported in the literature are listed in Supplementary Table 1, in which we reported the effect of the used treatments (PN or PLP administration) and the clinical outcome of the disease. Some variants have already been biochemically characterised, namely R95C, R116Q and R229W^8,15,19^. The most frequent mutation involves the Arg225 residue, which was found in 22 patients, each of whom showed different phenotypes, from mild^21,22^ to severe ones^21,23^; in one case this mutation was lethal^21^. In 16 patients, Arg225 was replaced with a histidine residue, in 4 with leucine, whereas two children presented with the R225C replacement. Moreover, in 7 cases the R225H replacement was found associated with R116Q. Given the high number of patients with mutation at the Arg225 residue, the R225H and the double R116Q/R225H PNPO variants were characterised. Among all known variants, two exhibiting a mild clinical outcome, G118R^24^ and R141C, were also chosen for characterization. The latter was found in one patient who also had a deletion of three amino acid residues (Ala94_Leu97del;^21^). This patient responded well to PN administration, showing a positive outcome. Transfection of CHO-K1 cells with the *PNPO* gene containing the R141C mutation resulted in 50% enzyme activity with respect to the wild type gene^21^. Finally, another mutation, which is characterised by the replacement of the stop codon with a glutamine residue (X262Q), was analysed. This mutation, which leads to the formation of a longer protein (28 amino acids are added at the C-terminal end), resulted in a severe clinical outcome on patients treated with PN^13^.

### Physical properties of variant PNPO forms

**Figure 1:**
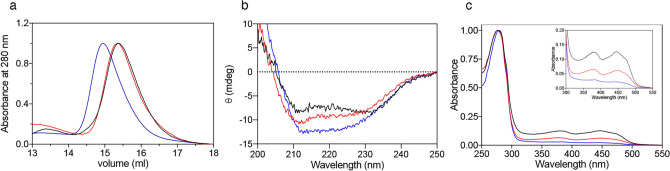
Physical properties of PNPO variant forms. Only results from the wild type (black lines), G118R (red lines) and X262Q (blue lines) PNPO enzymes are shown. All the other variants have the same characteristics as the wild type enzyme and therefore are not reported. (**a**) Size exclusion chromatography analyses on PNPO variants. Elution profiles were obtained in 20 mM potassium-phosphate buffer, pH 7.6, containing 150 mM NaCl and 5 mM 2-mercaptoethanol. Far-UV CD (**b**) and absorption (**c**) spectra of wild type, G118R and X262Q PNPO enzymes are shown. Inset in panel C is an expanded view of the visible region of the same absorption spectra, showing absorption bands due to FMN. For each protein sample three independent preparations were analysed, although in the figure a single spectrum is shown of wild type (71% saturation with respect to FMN), G118R (30% saturation) and X262Q (10% saturation). All spectra were measured in 20 mM potassium-phosphate buffer, pH 7.6. Images were generated using the software Prism 8 (GraphPad; https://www.graphpad.com/scientific-software/prism/).

The G118R, R141C, R225H, R116Q/R225H, and X262Q PNPO variants were produced, expressed and purified following the protocol used for the wild type enzyme (described in Methods). Size-exclusion chromatography showed variant and wild type enzymes to have similar elution profiles (Fig. 1a), corresponding to the dimeric form of the protein. The X262Q variant showed a shift in the elution profile, compatible with the higher molecular weight of the protein due to the insertion of 28 amino acid residues. CD spectra of all variants, except those of G118R and X262Q variants, also showed no significant secondary structure differences compared to wild type (Fig S1A). The CD spectrum of the X262Q variant was slightly different, according to the presence of an extra sequence at the C-terminal end. In the G118R variant, the replacement of the small glycine residue with an arginine residue may have altered the α2-helix of PNPO (Fig. 1b). These data suggest that the replacements did not substantially affect neither the oligomerization state nor the overall structural integrity of the enzyme.

The amount of FMN bound to the variant forms was quantified on the basis of the absorbance at 445 nm^25^ measured at the end of the purification procedure (Fig. 1c and Supplementary Fig. S1B). The wild type enzyme, the R225H and R116Q/R225H variants were about 77% saturated by FMN with respect to protein subunits (Table 1). The R141C variant contained a slightly lower amount of FMN, resulting in 57 ± 10% saturation (Supplementary Fig. S1B). On the other hands, the G118R form contained 44 ± 6% FMN with respect to the protein content, whereas only 22 ± 6% X262Q was saturated with the cofactor (Fig. 1c; Table 1). The dissociation constant of the FMN binding equilibrium with the G118R, R141C, and X262Q variants was measured performing fluorescence titration experiments. The apo-form of the enzymes was produced as explained in^15^. The excitation was set at 450 nm and the emission fluorescence was recorded between 470 and 570 nm. Data were analysed as previously reported^26^. The G118R variant binds the cofactor with a dissociation constant (94 ± 19 nM) that is about sevenfold higher as compared with that of the wild type enzyme (13.1 ± 1.7 nM;^19^), whereas for the R141C and X262Q the K_D_ values were estimated to be much higher, i.e. 1.3 ± 0.3 μM and 2.4 ± 0.4 μM, respectively (Table 1 and Supplementary Fig. S2).

**Figure 2:**
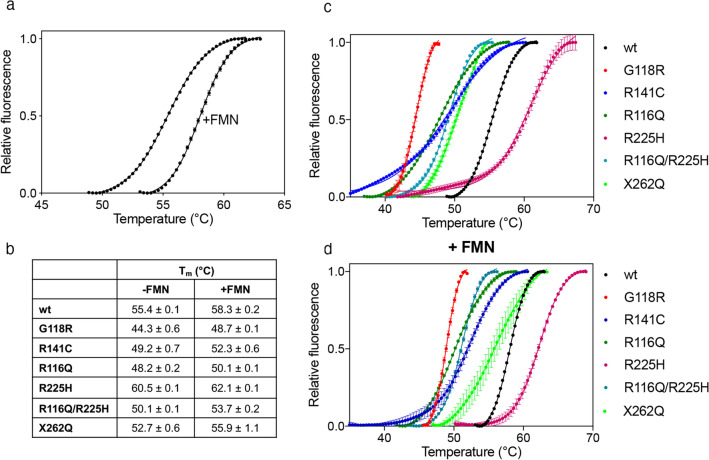
Differential scanning fluorimetry measurements with variant PNPO forms*.* (**a**) Fluorescence change, expressed as fractional variation as a function of temperature of 2 μM wild type PNPO as purified and in the presence of an equimolar amount of FMN. The Boltzmann equation was fitted to data, obtaining the melting temperatures listed in (**b**). Fluorescence change, expressed as fractional variation as a function of temperature of wild type PNPO and all variants indicated in the legend, as purified (**c**) and in the presence of an equimolar amount of FMN (**d**). The curves shown in the figure are the average with standard error bars of three independent experiments. Images were generated using the software Prism 8 (GraphPad; https://www.graphpad.com/scientific-software/prism/).

To verify if the mutations affect the thermal stability of the encoded proteins, which may contribute to the onset of the disease, all PNPO variants described above were analysed using Differential Scanning Fluorimetry (DSF) technique (Fig. 2). Experiments employed purified wild type and variant PNPO forms as obtained from the purification procedure and after the addition of an equimolar amount of exogenous FMN (2 μM), which had the purpose to saturate all enzyme forms with cofactor. However, because of an intrinsic limit of the technique (see Methods), it was possible to add a maximum of 2 μM FMN, which corresponded to an equimolar amount with respect to the enzyme. In the case of the R141C and X262Q variants, this concentration of FMN was not enough to saturate the enzyme. However, it is clear that the presence of the cofactor stabilizes all the PNPO forms by increasing the melting temperature (T_m_) by 2–4 °C. Whether in the presence of FMN or not, all variants are less stable than the wild type (T_m_ = 55.4 ± 0.1 °C), except the R225H variant (T_m_ = 60.5 ± 0.1 °C), which is more stable. It can be predicted that the R141C and X262Q variants would have a higher T_m_ when completely saturated with FMN, which could match the value obtained with wild type PNPO. Furthermore, the already characterized R116Q variant^19^ was also analysed in comparison to the double R116Q/R225H PNPO variant. Concerning the R116Q variant, the T_m_ is 48.2 ± 0.2 °C, which is lower than wild type. Similarly, the double variant R116Q/R225H displays a T_m_ of 50.1 ± 0.1 °C. R141C and X262Q variants are slightly less stable than the wild type, showing T_m_ values of 49.2 ± 0.7 °C and 52.7 ± 0.6 °C, respectively. The less stable variant is G118R, which has a T_m_ = 44.3 ± 0.6 °C, which increases to 48.7 ± 0.1 °C in the presence of FMN (Fig. 2b).

### Kinetic properties of PNPO variants

Kinetic measurements for the PNPO variants, as for wild type, were carried out by following the formation of the aldimine complex between PLP and TRIS, which maximally absorbs at 414 nm, using 50 mM TRIS–HCl buffer pH 7.6, at 37 °C (Supplementary Fig. S3). The resulting K_M_ and k_CAT_ values of wild type and variant forms (Table 1) were determined using PNP as substrate, and analysing the initial velocity data with the quadratic Eq. (2), since low substrate concentrations and a relatively high enzyme concentration (2 μM) had to be used, as explained in Methods section. In the case of the G118R and X262Q variants, which after purification were not fully saturated with FMN, a molar excess of cofactor was added to the reaction mixture (6.25 μM, about 3 times the enzyme concentration, which was 2 μM). However, we could not measure any activity with the X262Q variant, even by increasing the FMN concentration to 200 μM and the enzyme concentration up to 5 μM. This suggests that the addition of 28 amino acid residues to the C-terminal end of the protein has a large impact on the enzyme functionality. All other replacements increased K_M_ values and decreased the k_CAT_. Among these, the R225H variant showed the most drastic change of kinetic parameters with respect to wild type, having a 27-fold lower k_CAT_ and sixfold higher K_M_. Remarkably, the addition of the R116Q replacement to the R225H single variant improves catalytic efficiency. In fact, with respect to the single R225H variant, the K_M_ of the R116Q/R225H double variant is 3 times lower, whereas the k_CAT_ is 6 times higher. Kinetic parameters of the recombinant enzymes obtained in our study account for the percentage of activity measured by other authors under fixed conditions^12,13,21^ (Table 1).

### Allosteric regulation of human PNPO

In previous studies carried out on recombinant human and *E. coli* PNPO, it was shown that these enzymes are inhibited by the reaction product, PLP, and it was proposed that this inhibition is caused by PLP binding at the active site. The same authors suggested that PLP also binds tightly at a secondary site, distinct from the active site, apparently without causing inhibition^6,27^. However, as stated in the introduction, we have recently demonstrated that PLP inhibits *E. coli* PNPO by binding at the secondary site, and therefore that PLP inhibition is of an allosteric nature^7^. Here we hypothesised that an allosteric PLP binding site, that controls the enzyme activity, might also be present in human PNPO. To test this hypothesis, we carried out a series of kinetics measurements with human PNPO, and observed a behaviour similar to that of the *E. coli* enzyme.

**Figure 3:**
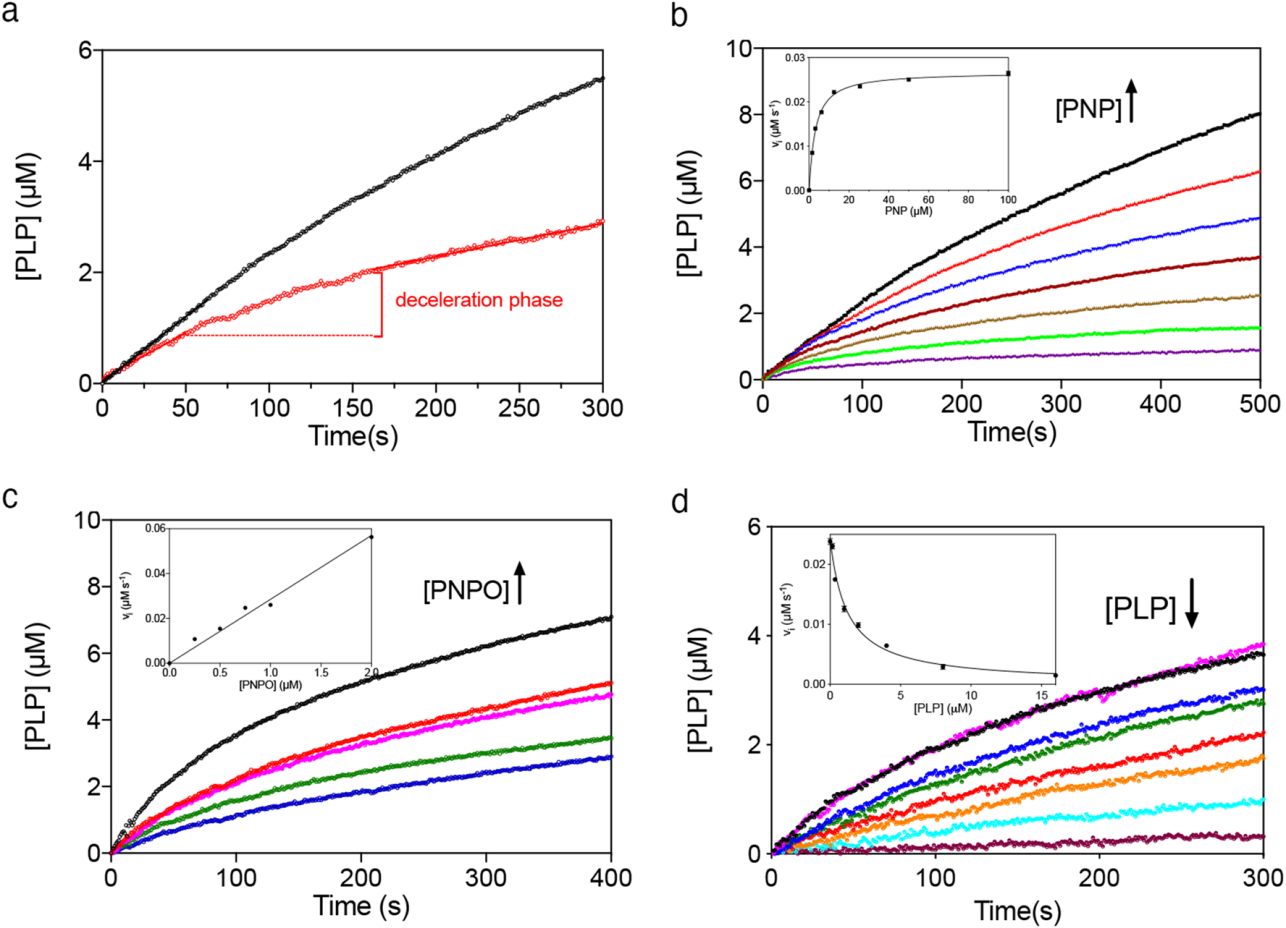
Analysis of reactions catalysed by PNPO in HEPES buffer. (**a**) Kinetics of PNP oxidation to PLP in TRIS and HEPES buffers catalysed by wild type PNPO. Kinetics were carried out with 0.5 μM enzyme (protein subunit concentration) and 15 μM PNP, in 50 mM TRIS–HCl and 50 mM Na-HEPES buffers at pH 7.6, containing 5 mM 2-mercaptoethanol. The black and red dashed lines indicate the linear production of PLP observed in TRIS buffer over the first 100 s of reaction, and the linear PLP production that follows the deceleration phase observed in HEPES buffer, respectively. (**b**) A fixed concentration of enzyme (0.5 µM) was mixed with buffer containing different PNP concentrations (1.56, 3.13, 6.25, 12.5, 25, 50 and 100 µM). Kinetic traces obtained with increasing PNP concentrations, as indicated by the arrow, are displayed in different colours. The inset shows the initial velocity of the reaction, calculated by fitting the first 20 s of the kinetics to a linear equation, as a function of substrate concentration. This saturation curve was analysed using the Michaelis–Menten equation, obtaining the kinetic parameters listed in the text. (**c**) Increasing enzyme concentration (0.25, 0.5, 0.75, 1 and 2 µM, as indicated by the pointing up arrow), while keeping PNP concentration fixed (15 µM), proportionally increased the initial velocity of the reaction, as shown in the inset. (**d**) Effect of PLP on reaction kinetics in HEPES buffer. Kinetics obtained by the addition of increasing concentrations of exogenous PLP (0, 0.17, 0.35, 1, 2, 4, 8 and 16 µM, as indicated by the pointing down arrow) to reactions containing 0.5 µM enzyme and 15 µM PNP. The inset shows the initial velocity of the reaction as a function of PLP concentration, fitted to a decreasing hyperbolic equation. Images were generated using the software Prism 8 (GraphPad; https://www.graphpad.com/scientific-software/prism/).

**Figure 4:**
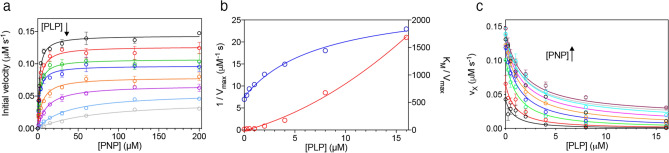
Characterization of PLP inhibition. (**a**) The initial velocity of the reaction was measured with 2 µM enzyme (protein subunit concentration), varying PNP concentration while keeping exogenous PLP fixed at different concentrations (0, 0.25, 0.5, 1, 2, 4, 8 and 16 µM, as indicated by the pointing down arrow). Data are the average ± standard deviation of three independent measurements. The Michaelis–Menten equation was fitted to the resulting saturation curves, obtaining apparent V_max_ and K_M_ values at all PLP concentrations. (**b**) Fitting of 1/V_max_ (blue symbols) and K_M_/V_max_ (red symbols), obtained from the fitting of data shown in panel A, using an increasing hyperbolic equation and a parabolic equation, respectively. (**c**) Initial velocity data rearranged as a function of PLP concentration, and obtained at different, fixed substrate concentrations (v_X_). The increasing PNP concentration is indicated by the pointing up arrow. Continuous lines result from global fitting of v_x_ values to Eq. (3), as detailed in the Methods section, which gave the parameters reported in the text. Images were generated using the software Prism 8 (GraphPad; https://www.graphpad.com/scientific-software/prism/).

**Figure 5:**
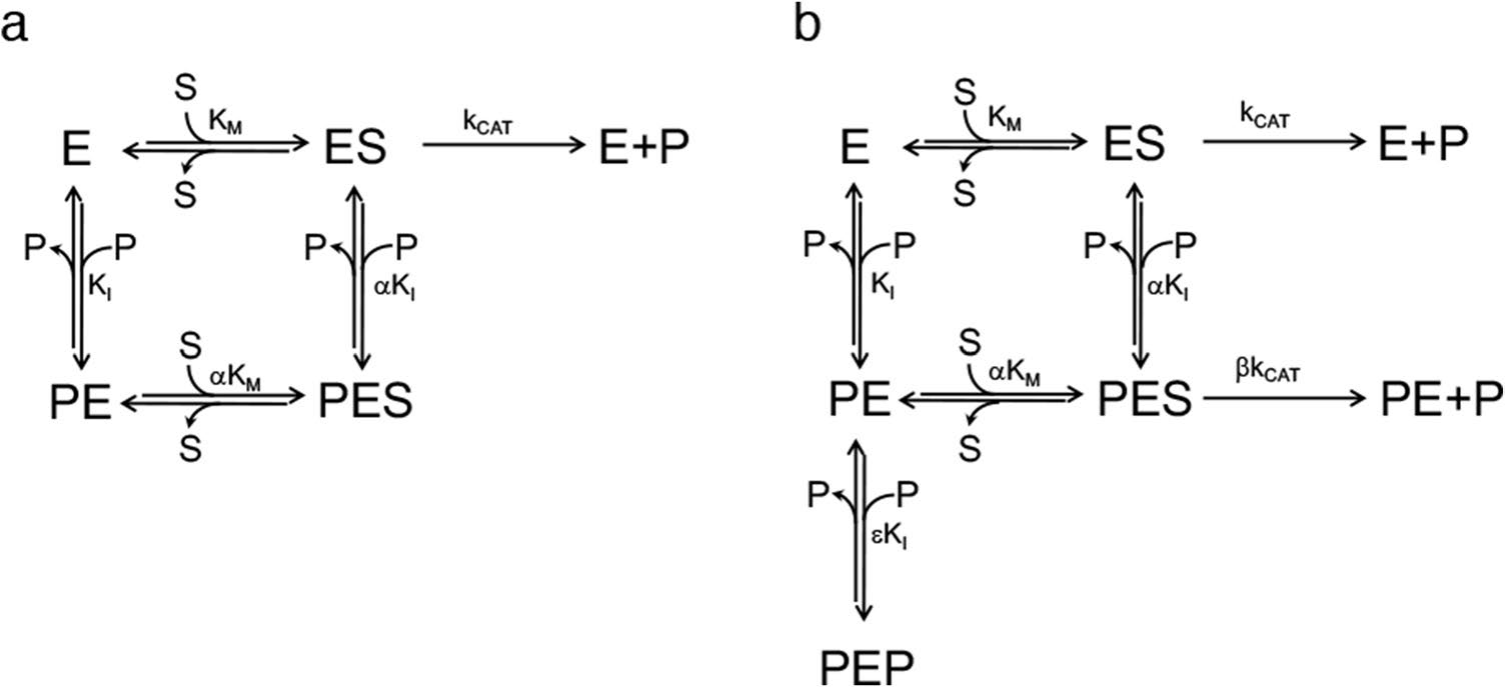
(**a**) Steady-state kinetics model of the linear mixed-type inhibition demonstrated in the case of *E. coli* PNPO^7^, in which the enzyme (E) is able to bind both the PNP substrate (S) and the PLP product (P) at the same time, respectively at the active site and at an allosteric site. Binding of substrate and PLP affect each other by increasing K_I_ and K_M_ of a α coefficient. The PES ternary complex is catalytically inactive. (**b**) Steady-state kinetics two-sites mixed modification model corresponding to the parabolic inhibition observed with human PNPO. This model is similar to that shown in the previous panel; however, in this case, the PES ternary complex is catalytically active, although k_CAT_ is reduced by a β coefficient. Moreover, the PE complex, formed upon binding of PLP at the allosteric site, is able to bind a second PLP molecule at the active site (forming the dead-end PEP complex) with a K_I_ that is increased of a ε coefficient.

First of all, the PNP oxidation reaction catalysed by wild type human PNPO was analysed in either Na-HEPES or TRIS–HCl buffer. It should be considered that in TRIS buffer, as PLP is produced in the reaction catalysed by PNPO, it forms a Schiff base with the amino group of TRIS and is sequestered from the solvent. This does not happen in HEPES buffer, where PLP is free to accumulate in the solvent. As seen in Fig. 3a, PLP formation kinetics is different when measured with 15 μM PNP substrate in TRIS and HEPES buffer. In TRIS buffer, the rate of PLP formation keeps constant over about 100 s, after which it starts decreasing. On the other hand, in HEPES buffer although over the first few seconds (i.e. when PLP has not yet accumulated to a great extent) PLP formation shows a linear course, over a longer time extent a neat deceleration phase is observed, followed by a second phase that appears approximately linear over the considered time interval. This behaviour is evident at all substrate concentrations (Fig. 3b). The initial velocity of the reaction at different PNP concentrations was measured by linear fitting of the first 20 s of the kinetic traces. The inset in Fig. 3b shows that the initial velocity of the reaction increases hyperbolically as a function of PNP. Fitting this saturation curve to the Michaelis–Menten equation gave kinetic parameters K_M_ = 2.8 ± 0.2 μM and k_CAT_ = 0.057 ± 0.002 s^−1^, which are in good agreement with those reported in the literature and determined in TRIS buffer^6,19^. The kinetics at different PNP concentrations were also analysed in TRIS buffer according to the same procedure, obtaining values of K_M_ = 2.6 ± 0.2 μM and k_CAT_ = 0.062 ± 0.002 s^−1^ (Table 1). Furthermore, when the experiment was performed in HEPES buffer with a fixed amount of substrate (15 µM) and increasing enzyme concentrations (from 0.25 to 2 µM) (Fig. 3c), the amplitude of the deceleration phase grew with decreasing enzyme concentration and the initial velocity of the reaction increased proportionally to that concentration (Fig. 3c, inset). To check if the increase of PLP concentration influences the time course of the reaction, different exogenous PLP concentrations (from 0.17 to 16 μM) were added to reaction mixtures containing 0.5 μM PNPO and 15 μM PNP (Fig. 3d). In this case, as PLP concentration was increased, the initial velocity of the reaction decreased hyperbolically (Fig. 3d, inset) and the deceleration phase of the reaction tended to disappear with increasing concentration of added PLP. The features of data shown in Fig. 3 are very similar to those observed with the *E. coli* PNPO^7^. Moreover, the deceleration phase of reaction kinetics, followed by a linear phase, observed in HEPES buffer are clearly not compatible with a competitive inhibition mechanism. Thus, a complete inhibition kinetics characterisation with human PNPO was carried out in HEPES buffer. The initial velocity of the reaction was measured varying PNP concentration at different, fixed exogenous PLP concentrations, using 2 μM enzyme (Fig. 4a). Because of the high enzyme concentration, the quadratic Eq. (2) should be fitted to saturation curves, as explained above (see Methods). However, because our following data analysis requires treatment of data according to the Michaelis–Menten equation, we used this to calculate kinetic parameters. This is a good approximation, as demonstrated by the similar values of kinetic parameters obtained fitting data to either the quadratic equation of the Michaelis–Menten equation. The analysis of saturation curves demonstrated that PLP inhibition of human PNPO, like that of the *E. coli* enzyme, is not competitive but has a mixed type nature. However, the secondary plots of 1/V_max_ and K_M_/V_max_ as a function of PLP concentration (Fig. 4b) reveal that the inhibition mechanism is not the same observed in the case of *E. coli* PNPO (scheme in Fig. 5a), in which such plots are both linear^7^. With human PNPO, 1/V_max_ shows a hyperbolic increase as a function of PLP concentration, whereas K_M_/V_max_ shows a parabolic increase. This peculiar behaviour is characteristic of parabolic inhibition (the term parabolic refers to the peculiar shape of the K_M_/V_max_ plot), which corresponds to a two-sites mixed modification model, such as that shown in Fig. 5b^28^ (also visit https://www.enzyme-modifier.ch/parabolic-inhibition/). According to this inhibition mechanism, two molecules of the same “modifier” (in our case PLP) bind at two different sites on the enzyme: an allosteric site and the active site. Binding of PLP at the active site leads to a dead-end complex. On the other hand, binding of PLP at the allosteric site forms a binary complex that is still able to bind the substrate (although with reduced affinity with respect to the free enzyme), resulting in a productive ternary complex, in which the substrate is converted to product (although with reduced k_CAT_ with respect to the enzyme–substrate complex) (see Fig. 5b legend for details). The model depicted in Fig. 5b assumes a sequential binding of PLP to the enzyme, by which PLP first binds to the allosteric site of the free enzyme (forming PE) and then to the active site (forming PEP). A more complex, general model can be envisaged in which the PEP dead end complex is formed randomly, so that PLP can also bind to the active site of the free enzyme (forming EP) and then to the allosteric site (forming PEP). We decided to apply the simplified model of Fig. 5b since it allows an easier analysis of data and does not make any difference with respect to the main feature of the system, i.e. the capability of PNPO to bind two molecules of PLP. This choice is justified by the results of kinetic simulations that we carried out using either the general model or the simplified model, which were similar (data not shown). The data shown in Fig. 4a were rearranged as the initial velocity measured at variable PLP concentration at a fixed substrate concentration (v_X_). Equation (3)^28^ (see Methods) was fitted to the v_x_ values at all different substrate concentrations, assuming rapid equilibrium binding of both substrate (PNP) and modifier (PLP), with shared parameters, whereas K_M_ was kept fixed at the value determined in the absence of PLP (1.97 μM). The parameters obtained from the fitting are K_I_ = 0.62 ± 0.09 μM, α = 2.72 ± 0.65, β = 0.15 ± 0.04 and ε = 6.57 ± 4.73.

### Analysis of PLP binding equilibrium

**Figure 6:**
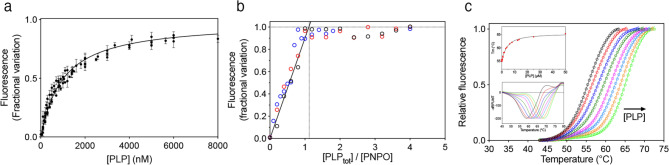
Analysis of PLP binding equilibrium of wild type PNPO. (**a**) The figure shows the PLP binding curve obtained with 100 nM PNPO (protein subunit concentration). Reported data are the average ± standard deviation of five independent measurements. Equation (4) (see Methods) was fitted to these data. (**b**) Binding stoichiometry analysis obtained with 1 μM (black symbols), 2 μM (red symbols) and 4 μM (blue symbols) protein subunit concentrations. Fluorescence change, expressed as fractional variation as a function of the [PLP_tot_]/[PNPO] ratio, is linear as shown by the continuous line, up to the stoichiometry point corresponding to the crossing with the horizontal dotted line. The vertical dotted line through the stoichiometry point indicates that about one PLP molecules binds per enzyme subunit. (**c**) DSF measurements with wild type PNPO in the presence of different PLP concentrations*.* Fluorescence change is expressed as fractional variation as a function of temperature. The experiment was carried out using 2 µM enzyme and increasing PLP concentrations (0, 0.78, 1.56, 3.13, 6.25, 12.5, 25 and 50 µM, as indicated by the arrow). The Boltzmann equation was fitted to thermal denaturation data to obtain melting temperatures. Each curve is the average of three independent experiments but, to clearly show the data, the standard error bars were not reported in the graph. In the upper inset, the saturation curve obtained by plotting the melting temperatures as a function of the PLP concentrations is shown. Data were analysed using the quadratic Eq. (5) (see Methods) to estimate the dissociation constant reported in Table 1. In the lower inset, the graph shows the first derivative (–dRFU/dT) of the fluorescence emission data reported in panel C. Images were generated using the software Prism 8 (GraphPad; https://www.graphpad.com/scientific-software/prism/).

The kinetic analysis described in the previous paragraph suggested that PLP binds at both the active site and at an allosteric site on human PNPO. Two different fluorimetric methods were used to obtain a direct measurement of PLP binding to human PNPO. The first one took advantage of the increase of FMN fluorescence emission observed upon binding of PLP to the protein^7^. The experiment was performed by mixing increasing PLP concentrations (from 100 to 8,000 nM) with 100 nM enzyme, in 50 mM Na-HEPES buffer pH 7.6, at 25 °C. The measured increase of fluorescence as a function of PLP concentration was analysed using a quadratic equation (Eq. (4) in Methods), which allowed to estimate the dissociation constant of the PLP binding equilibrium and that we are going to define as K_D1_. The determined K_D1_ value of 0.98 ± 0.02 µM (Fig. 6a) is comparable to the K_I_ for PLP binding at the allosteric site of the free enzyme determined by inhibition kinetics (Fig. 5b). Then, using the same fluorimetric method, more concentrated PNPO solutions (1, 2 and 4 µM) were titrated with PLP in order to determine the stoichiometry of PLP binding, which was found to be of two molecules of PLP per PNPO dimer (Fig. 6b).

Moreover, the binding of PLP to human PNPO was also analysed using the DSF. When PNPO (2 µM) was heated in presence of different PLP concentrations, from 0.78 to 50 µM, the thermal stability of the protein increased (Fig. 6c). The estimated melting temperatures, resulting from fitting of data to the Boltzmann equation^29^, were plotted as a function of PLP concentration. The resulting saturation curve (Fig. 6c inset) was analysed using the quadratic Eq. (4) (see Methods) and gave a dissociation constant (K_D2_) of 3.2 ± 0.2 µM. Interestingly, this value is comparable to εK_I_ (6.57 × 0.62 μM = 4.07 μM) determined from PLP inhibition kinetics and corresponding to PLP binding at the active site (Fig. 5b).

### PLP binding and allosteric inhibition in PNPO variants

**Figure 7:**
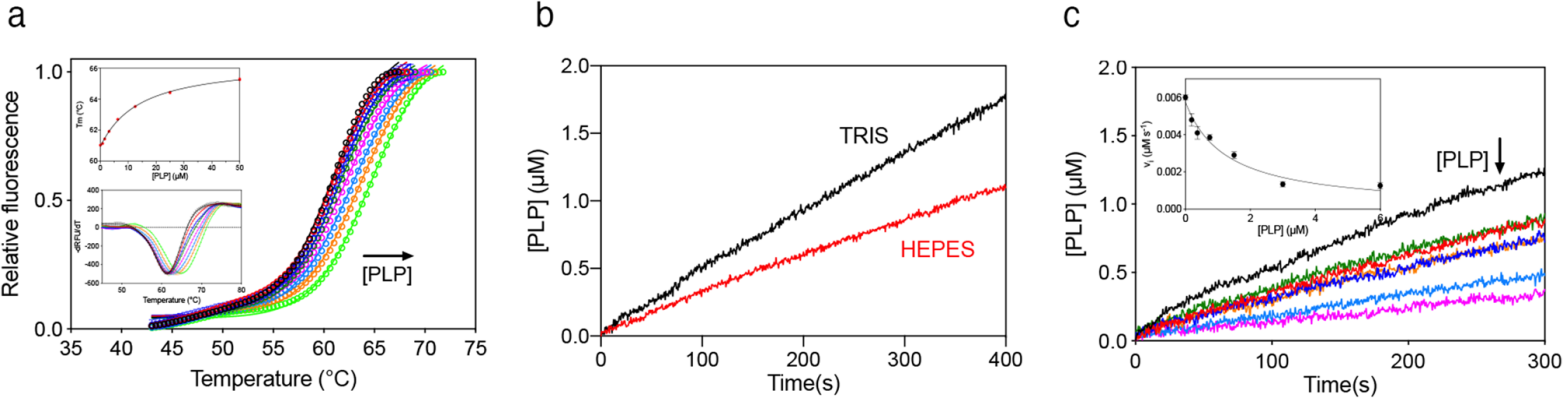
PLP binding and allosteric properties of the PNPO R225H variant*.* (**a**) Fluorescence change expressed as fractional variation is plotted as a function of temperature. The experimental conditions were the same as those reported in Fig. 6c. The curves are the result of three independent experiments but, to better show the data, the standard error bars were not reported in the graph. In the upper inset, the saturation curve obtained by plotting the melting temperatures as a function of the PLP concentrations is shown. Data were analysed using the quadratic Eq. (4) (see Methods) to estimate the dissociation constant reported in Table 1. In the lower inset, first derivative (–dRFU/dT) of the same data reported in panel A. Kinetic studies of R225H variant. (**b**) Comparison of kinetics carried out in 50 mM TRIS–HCl and 50 mM Na-HEPES buffers at pH 7.6, obtained using 0.5 μM enzyme and 60 μM PNP. (**c**) Kinetics obtained by the addition of increasing concentrations of exogenous PLP (0, 0.19, 0.38, 0.75, 1.5, 3 and 6 µM, as indicated by the pointing down arrow) to reactions. The experiments were carried out with 1 μM enzyme and 60 μM PNP. A decreasing hyperbolic equation was fitted to the initial velocity of the reaction as a function of PLP concentration (inset). Images were generated using the software Prism 8 (GraphPad; https://www.graphpad.com/scientific-software/prism/).

The two fluorimetric methods used to determine PLP binding to wild type PNPO were also applied to the G118R, R141C, R225H, R116Q/R225H, and X262Q variants (Supplementary Figs S4 and S5); the calculated K_D1_ and K_D2_ values are reported in Table 1. Concerning the PLP high affinity binding site, the K_D1_ values measured using FMN fluorescence emission were the same as that of wild type PNPO for all variants, except for G118R, whose K_D1_ was lower (0.26 ± 0.01 μM). With all variants, DSF measurements revealed an increase of thermal stability upon binding of PLP, except in the case of the G118R and X262Q variants, for which the addition of PLP had no effect on the melting temperature (Fig. 7a and Supplementary Fig. S5). As with wild type PNPO, the increase of T_m_ values as a function of PLP concentration showed a hyperbolic behaviour, allowing estimate of a dissociation constant (Table 1). These K_D2_ values were in the range of that of the wild type enzyme, except in the case of the R225H variant, which showed a higher value (Table 1). The ability of all variants to bind PLP at the high affinity binding site suggests that they have maintained the allosteric inhibition observed in the wild type enzyme. To confirm this hypothesis, the kinetics of PLP formation obtained in TRIS buffer was compared with that observed in HEPES buffer with all variants, except with the X262Q variant, which has no activity (Fig. 7b and Supplementary Fig. S6). The results clearly show that, in all cases, PLP accumulation in HEPES buffer determines an inhibition of the enzyme activity, as described before for the wild type enzyme. A further kinetic characterisation was performed with the R225H PNPO variant. In particular, we observed that, as for wild type PNPO (Fig. 3d), adding exogenous PLP (from 0.19 to 6 μM) in HEPES buffer (Fig. 7c) makes the first deceleration phase disappear.

## Discussion

To date, 15 different missense mutations have been reported in PNPOD-affected patients. However, only 3 have been characterized in vitro with respect to the functional and structural properties of the recombinantly expressed protein^8,15,19^. Seizures usually appear in the first week of life and cease upon treatment with PLP. Nevertheless, the identification of new mutations has changed the symptomatology of the illness. For instance, the age of seizure onset increases up to 3 years in children homozygous for the R116Q variant^19^; in other cases switching of treatment from PN to PLP have caused the recurrence of seizures^12,21^. For these reasons, the knowledge of the molecular bases of the disease is important, and could allow a more rational use of treatment. To precisely understand how the pathogenic replacements G118R, R141C, R225H, R116Q/R225H, and X262Q affect PNP oxidase enzymatic activity, these variant forms were recombinantly produced and characterized.

The G118 residue is highly conserved (Supplementary Fig. S7) and located at the C-terminal end of the α2-helix that electrostatically interacts with the phosphate group of FMN (Supplementary Fig. S8). The G118R replacement affects FMN binding, as shown by the low percentage of this cofactor bound to the purified protein (44% with respect to protein subunits) and the higher K_D_ for the FMN binding equilibrium (94 nM, compared to the 13 nM value determined with wild type PNPO). This observation suggests that the G118R replacement has a perturbing effect on the α2-helix structure. This hypothesis is supported by the much lower melting temperature measured with this variant (Fig. 2) and the altered far-UV CD spectrum (Fig. 1b). Such a structural alteration also affects the catalytic properties of the variant, which shows a higher K_M_ and a lower k_CAT_.

R141C was found only in 1 patient, associated with deletion p.A94_L97del (Supplementary Table 1). The transfection of CHO-K1 cells with DNA containing only the p.Arg141Cys mutation resulted in the expression of an enzyme with reduced activity with respect to wild-type PNPO^21^, suggesting that this mutation affects the enzyme function. Residue Arg141 is one of the 14 amino acids that are involved in direct or water-mediated intersubunit interactions with FMN, that is bound at the dimer interface^6, 15^ (Supplementary Fig. S8). These interactions stabilize FMN binding, and probably ensure a correct FMN orientation for optimal substrate oxidation. Expectedly, the R141C variant binds the cofactor with a dissociation constant that is ∼100-fold higher than that of the wild type enzyme, and shows a k_CAT_ more than 3 times lower and a K_M_ about 2 times higher than wild type PNPO (Table 1).

**Figure 8:**
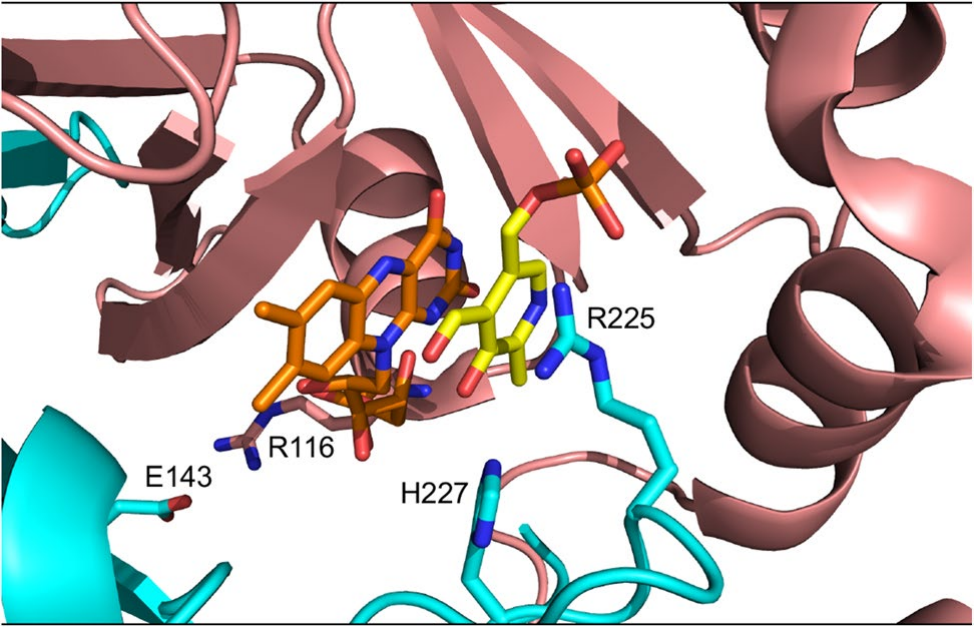
Close-up view of the active site of human PNPO. Crystallographic structure obtained in the presence of PLP (PDB: 1NRG;^6^). FMN and PLP in the active site are shown as sticks, in orange and yellow colour, respectively. The R225, R227, R116, and E143 residues are shown as sticks. The image was created using PyMOL v1.7.4.5 Edu Enhanced (https://pymol.org/2/).

Arg225 is highly conserved from bacteria to humans (Supplementary Fig. S7) and it had been suggested to contribute to substrate binding and orientation (Fig. 8). For a correct and efficient catalysis, the PNP pyridine ring must be placed parallel to the FMN isoalloxazine ring. In the wild type structure, there are very close hydrophobic contacts between the substrate pyridine ring and the FMN isoalloxazine ring, as well as hydrogen bond interactions between the strictly conserved residues His227 and Arg225 and the substrate^15^. The side chain of the latter residue forms a salt bridge interaction with the phosphate group of PNP. Moreover, in a study with the *E. coli* enzyme, the corresponding Arg residue (Arg197), which is located at about 3.4 Å from the C4′ carbon of PNP, has also been shown to play an important role in controlling the stereospecific transfer of the pro-R hydrogen of the substrate to FMN. The substitution with a Glu residue resulted in a 8,000-fold increase and a 16-fold decrease in K_M_ and k_CAT_, respectively, when PNP is used as substrate^4^. Also, a previous study on HeLa cells expressing the human enzyme showed that the R225C and R225H replacements resulted in a 91% and 92% reduction in the amount of PLP synthesized over a 40-min period by PNPO, as compared to the wild type enzyme, although in this case PMP was used as substrate^12^. However, it should be noticed that with human PNPO the kinetic parameters obtained with PMP as substrate are very similar to those obtained with PNP^6^. Ten patients have been found to be homozygous for the R225H mutation^21–23^, 1 for the R225C mutation^30^ and 4 for the R225L mutation^31^. Our kinetic characterisation of the R225H variant showed a very low k_CAT_ value and a much higher K_M_, indicating that this residue is of fundamental importance for the catalysis of the reaction. Interestingly, the melting temperature of this variant is higher than that measured with the wild type PNPO, whereas all the other variants show a lower T_m_. In the active site structure of PNPO, the positive charge of Arg225 is not counterbalanced by a close interaction with any other residue (Fig. 8). Therefore, Arg225 may represent a destabilizing residue and the increased thermal stability of the R225H variant may result from the loss of its unbalanced positive charge. There are other examples of residues involved in catalysis whose replacement leads to a more stable but less active enzyme^32,33^.

In 7 patients, R225H is associated with the R116Q variant (Supplementary Table 1), and in 3 such cases when the treatment with PN was switched to PLP, a recurrence of seizures was observed^12,21^. Our results with the R116Q/R225H double variant show that the addition of the R116Q variant has the effect to considerably ameliorate the negative consequences of the R225H replacement on the catalytic properties (Table 1). The Arg116 residue is involved in an electrostatic interaction with Glu143 from the other subunit of the dimer^19^. Since the residues that form the active site of PNPO are contributed by both subunits, the R116Q replacement might affect this microenvironment, improving substrate binding and catalysis compromised by the R225H replacement. The effect of the additional R116Q variant on the enzyme structure is made evident by the decrease of T_m_ of the double variant, which is much lower than that of the R225H single variant and higher than that of the single R116Q variant (Fig. 2).

The homozygous mutation X262Q was responsible for the death of two siblings that showed PNPOD symptoms within 30 min from birth. PN administration was ineffective and both children died in a few weeks. PLP was not administered^13^. Our data on the recombinant enzyme confirm that this mutation, although has no effect on the quaternary structure of the protein, has a disruptive effect on the enzyme function, and is actually the most drastic one among those we have characterized. In fact, the recombinant X262Q variant was almost devoid of FMN, had a much higher K_D_ for FMN binding and was catalytically inactive. The drastic effect of this mutation on catalysis is explained if we consider that the C-terminus of PNPO is very close to FMN^6^. Since the X262Q mutation suppresses the stop codon and results in the translation of an over-sized C-terminal end, this probably protrude into the active site and may interfere with FMN and substrate binding (Supplementary Fig. S8).

PLP inhibition kinetics obtained with human PNPO reveal a mixed-type inhibition system (Fig. 4a). Given that PNPO follows a substituted-enzyme mechanism when acting on PNP as substrate (a ping-pong mechanism in which a hydride ion is first transferred from PNP to FMN and then from this cofactor to an oxygen molecule^4^), this inhibition pattern may be thought to derive from PLP binding at the active site of the reduced enzyme form (with FMNH_2_). However, as we have already argued in detail in the case of the *E. coli* enzyme^7^, for several reasons this hypothesis must be ruled out. The most convincing one is the fact that a mixed-type inhibition resulting from a ping-pong mechanism would not yield biphasic kinetics as we observed (Fig. 3), but a progressive decrease of reaction velocity as substrate is consumed in the course of the reaction, as in simple steady-state kinetics. Therefore, our kinetic characterisation of wild type human PNPO strongly suggests that, similarly to *E. coli* PNPO^7^, PLP inhibits the enzyme through an allosteric mechanism, although it revealed important specific features of the human enzyme. Moreover, it suggests that also in human PNPO the tight PLP binding site previously discovered by other authors^6^ corresponds to the allosteric site detected by our experiments. For human PNPO, we propose that PLP inhibition takes place according to the mechanism shown in Fig. 5b, whereas in the case of the *E. coli* enzyme the inhibition mechanism is that depicted in Fig. 5a. In both cases, the binding equilibria of substrate and inhibitor (PLP) to the enzyme are assumed to be rapidly established, and the conversion of the substrate into product to be relatively slow (this assumption, supported by experimental data with *E. coli* PNPO, may reasonably be extended to the human enzyme). Moreover, binding of substrate at the active site and binding of PLP at the allosteric site influence each other, increasing the respective dissociation constants (K_M_ and K_I_) by a α coefficient (which for human PNPO has a value of 2.72 ± 0.65, as estimated from data shown in Fig. 4c, whereas for *E. coli* PNPO is about 14^7^). However, while in the case of the *E. coli* enzyme, the ternary complex formed by the substrate bound at the active site and PLP bound at the allosteric site of the enzyme (PES in Fig. 5a, b) is completely inactive, in the human enzyme this complex retains the capability to catalyse the conversion of the substrate into product (this feature is responsible for the hyperbolic shape of the 1/V_MAX_ replot shown in Fig. 4^34^), although with a k_CAT_ that is reduced by a β coefficient (which has an estimated value of 0.15 ± 0.04). Moreover, human PNPO is able to bind PLP at the active site and at the allosteric site at the same time, forming the dead-end PEP ternary complex shown in Fig. 5b (which is responsible for the parabolic shape of K_M_/V_MAX_ replot^34^). In our previous experiments on *E. coli* PNPO, binding of PLP at the active site was not revealed by PLP inhibition kinetics (replots of 1/V_MAX_ and K_M_/V_MAX_ were linear), FMN fluorescence measurements (the increase of fluorescence as a function of PLP concentration indicated a single binding site, corresponding to the allosteric site, even at very high PLP concentration) and DSF measurements (PLP had no effect on the melting temperature of the protein, even at very high PLP concentration)^7^. At present, the location of the hypothetical allosteric PLP binding site on human PNPO is unknown. A crystallographic study on *E. coli* PNPO indicated the presence of a possible secondary PLP binding site on the protein surface, at a distance from the active site, in which PLP interacts with residues N84, K145 and F177A (*E. coli* PNPO numbering)^35^. Whether this secondary site actually corresponds to the tight PLP binding site has never been experimentally verified. The similar allosteric behaviour of the human and *E. coli* enzymes strongly suggests a similarity also in the structure and location of the allosteric PLP binding site. However, it should be noticed that two out of three of the residues composing the putative *E. coli* PNPO secondary site (K145 and F177 in *E. coli* PNPO) are not conserved in the human enzyme (Supplementary Fig. S7). In the crystal structure of human PNPO obtained in the presence of a large molar excess of PLP, this vitamer is located only at the active site of the enzyme, presumably with the same orientation assumed by the PNP substrate^6^. The reason why in these conditions PLP was not detected at the tight binding site is obscure. It is possible that the enzyme is able to assume alternative conformations, selected by the crystallization conditions, in which PLP is allowed or not allowed to bind at both the active site and the allosteric site at the same time.

Interestingly, although all characterised replacements affect the catalytic and FMN binding properties of human PNPO, PLP binding at the allosteric site is not altered by any of such replacements (Fig. 7 and Supplementary Fig. S6), except in the case of the G118R variant, which apparently binds PLP with a higher affinity than wild type PNPO (Table 1). This observation indicates that, evidently, none of the replaced residues is located at or is indirectly connected to the allosteric site. Moreover, since many of the replacements have strong effects on substrate binding and catalysis, this observation also confirms that PLP binding, revealed by the FMN fluorimetric measurements, is due to PLP binding at a secondary site, distinct from the active site. The exceptional case of the G118R variant may be attributed to a structural perturbation induced by the replacement in the α2-helix that somehow affects binding of PLP at both the allosteric and the active sites. A completely different picture is obtained when PLP binding is revealed by DSF measurements. In this case, with wild type PNPO, the measured K_D2_ is higher than the K_D1_ obtained with FMN fluorimetric measurements (and similar to εK_I_ obtained from inhibition kinetics), suggesting that it corresponds to PLP binding at a different site. Results obtained with the variant PNPO forms demonstrate that this site corresponds to the active site of the enzyme. In fact, K_D2_ values for PLP binding determined from DSF experiments with wild type and variant PNPO forms roughly correspond to K_M_ values determined from kinetic measurements (Table 1). In particular, the R225H variant, which shows a sixfold larger K_M_ with respect to wild type PNPO, also had a fivefold larger K_D2_ measured trough DSF experiments. These results on PLP binding at the active site also reinforce our interpretation of inhibition kinetics according to Fig. 5b, in which, differently from Fig. 5a that applies to the *E. coli* enzyme, PLP is able to bind both at the allosteric site and at the active site. Importantly, the X262Q variant, which has a heavily impaired active site, maintains unaltered the capability to bind PLP at the allosteric site. This suggests that it may still be able to bind PLP present in the environment and transfer it to apoenzymes, although very little or no PLP is expected to form inside the cell, given the very low catalytic activity of this PNPO variant.

An important difference between the human and the *E. coli* PNPO resides in the stoichiometry of PLP binding as revealed by FMN fluorescence measurements. In the bacterial enzyme, where PLP binding stoichiometry is of one molecule per protein dimer^7^, we have proposed that binding of PLP at one subunit might cause a conformational change that prevents binding at the second subunit. Since PLP binding stoichiometry with human PNPO is of two molecules per protein dimer (Fig. 6b), this mechanism of negative cooperativity may not be present in this enzyme. Alternatively, this stoichiometry may result from binding of a PLP molecule at the allosteric site on a monomer and of another PLP molecule at the active site of the other monomer.

It is worth noticing that most missense mutations in PNPOD concern codons that encode arginine residues (Arg95, Arg116, Arg141, Arg161, Arg225, Arg229; Supplementary Table 1). Among these residues, all except Arg225 are located in secondary structure elements of the protein. An investigation on the spectrum of disease-causing missense mutations in proteins showed that arginine is clearly the most replaced residue type^36^. Apparently, the very high mutability of arginine codons arises from the presence of CpG dinucleotides (arginine is coded by six codons, four of which have a CpG dinucleotide), which can spontaneously mutate by deamination either to TG or CA dinucleotides^37^. It is not surprising that in PNPO, replacements of arginine residues—interacting with the negative charges of FMN and PNP phosphate moieties—might affect cofactor and substrate binding, thermal stability, catalytic activity, and be responsible of several forms of PNPOD.

## Methods

### Site-directed mutagenesis, expression and purification of PNPO variant enzymes

All the variants were made using as template the cDNA coding for human PNPO, which had been cloned into the expression vector pET28a(+) (pET28-*PNPO*) as previously described^6^. Site-directed mutagenesis to obtain the G118R, R141C, R225H, and R116Q/R225H replacements was performed by QuickChange methodology (Stratagene, La Jolla). Mutagenic primer, synthesized by Metabion International AG (Steinkirchen, Germany), have the following sequences:

- G118R forward 5′-TCGAGAGTCGAAAAAGAAAAGAGCTGGA-3′,
- G118R reverse 5′-TCCAGCTCTTTTCTTTTTCGACTCTCGA-3′,
- R141C forward 5′-CTTAACCGTCAGGTGTGTGTGGAAGGCC-3′,
- R141C reverse 5′-GGCCTTCCACACACACCTGACGGTTAAG-3′,
- R225H forward 5′-GGTCAAACCAACCACCTGCATGACCGG-3′,
- R225H reverse 5′-CCGGTCATGCAGGTGGTTGGTTTGACC-3′,

with the nucleotides generating the mutation underlined. For the double variant R116Q/R225H, the mutagenic primers R225H forward and reverse were used on the pET28-*PNPO* construct expressing the R116Q variant^26^.

Concerning the X262Q variant, in which the stop codon was replaced with CAG (encoding a Gln residue), resulting in a 28-amino acid longer protein (as deduced from NCBI reference sequence NM_018129.4), seven PCR reactions were performed using the Platinum Super FI DNA Polymerase (Invitrogen), the standard T7 forward primer annealing on pET28 and the following reverse oligonucleotides:

- X262Q_1 5′-AGGTCCCAGAGCTGAGGTGCAAGTCTCTCATAG-3′
- X262Q_2 5′-CACTCTGGGCCAGCAGGTCCCAGAGCTGAGGT-3′
- X262Q_3 5′-ACCTAGCCCTAGCTCCACTCTGGGCCAGCAGGT-3′
- X262Q_4 5′-ACACCCTCTCTTGACACCTAGCCCTAGCTCCAC-3′
- X262Q_5 5′-CTGGGTCCCAATCCCACACCCTCTCTTGACACC-3′
- X262Q_6 5′-CTTAGAAAGAAGGGCCTGGGTCCCAATCCCACA-3′
- X262Q_7 5′-CCTTCGAATTCTTAGAAAGAAGGGCCTGGGTC-3′

In the first reaction, in which the pET28-*PNPO* was used as template and X262Q_1 as reverse primer, the stop codon was replaced with a Gln codon (underlined) and four new codons were added. The amplicon of this reaction was used as template for the subsequent reaction (which used the X262Q_2 as reverse primer) adding four new codons. This procedure was repeated 6 times using all the other primers. In the last reaction a new stop codon (double underling) and a restriction site for *Eco*RI was introduced. The last amplicon was digested with *Nde*I and *Eco*RI and inserted into pET28. All mutations were confirmed by sequence analysis (Microsynth AG, Balgach, Switzerland) of the constructs. Plasmids were purified using a NucleoSpin Plasmid kit from Macherey–Nagel.

Competent *E. coli* Rosetta(*λ*DE3) cells (Novagen) were transformed with pET28-*PNPO* constructs carrying the G118R, R141C, R225H, R116Q/R225H, or X262Q mutations. Transformants were grown in LB medium containing kanamycin (40 µg/ml) and chloramphenicol (34 µg/ml) to an OD_600_ of 0.8, induced with 0.2 mM isopropyl-β-d-thiogalactopyranoside, and incubated for 12 h at 28 °C. Cells were harvested, resuspended in 50 mM Tris–HCl pH 7.6, 300 mM NaCl, and ruptured by addition of lysozyme and sonication. After centrifugation, the expressed variant proteins were purified by metal-chelation chromatography, following the procedure described in^26^. Briefly, supernatant from cell lysis was directly loaded onto a 5 ml HisTrap FPLC column (GE Healthcare Life Sciences) and eluted with a 50 ml gradient of imidazole (from 0 to 300 mM) in resuspension buffer. Fractions containing the desired protein were pooled and dialyzed against 20 mM potassium phosphate, pH 7.8, containing 150 mM NaCl and 5 mM 2-mercaptoethanol. The apo-enzyme forms were prepared at low pH through a phenyl sepharose chromatographic step (GE Healthcare Life Sciences), as previously described^15^.

### Spectroscopic measurements

All spectra were acquired in in 20 mM potassium-phosphate pH 7.6 at 20 °C. UV–visible spectra were recorded using a Hewlett–Packard 8453 diode-array spectrophotometer, whereas a Jasco 710 spectropolarimeter equipped with a DP 520 processor was used to measure far-UV (190–250 nm) CD spectra, employing 0.1-cm path length quartz cuvettes.

### Size exclusion chromatography

Gel filtration of PNPO enzymes was performed on a Superdex 200 10/300 GL column (GE Healthcare) as previously described^26^.

### Measurement of the K_D_ of FMN binding

The dissociation constants for FMN binding to wild type and mutant enzymes were analysed taking advantage of FMN fluorescence quenching observed upon binding of the cofactor to apo-PNPO^6^. Apo-PNPO (from 5 nM to 4 μM) was added to FMN samples (50 nM) at 30 °C in 50 mM sodium HEPES buffer pH 7.6, 150 mM NaCl. Preliminary experiments demonstrated that, with all enzyme forms, the binding equilibrium was established within five minutes from mixing. Fluorescence emission spectra (470–570 nm; 4 nm emission slit) were recorded with a Horiba Jobin-Ivon FluoroMax-3 spectrofluorimeter, with excitation wavelength set at 450 nm (1 nm excitation slit), using a 1 cm path length quartz cell. Data were analysed according to Eq. (1), in which *F*_*rel*_ is the measured relative fluorescence at 525 nm, *F*_0_ is fluorescence in the absence of apo-PNP oxidase, *F*_*inf*_ is fluorescence at infinite apo-PNPO concentration, [*APO*] is the total apo-enzyme concentration, [*FMN*] stands for the total cofactor concentration and *K*_*d*_ is the dissociation constant of the equilibrium $APO + FMN \rightleftharpoons HOLO$

1 $$F_{rel} = F_{0} - \left( {F_{0} - F_{\inf } } \right) \times \frac{{\frac{{\left[ {APO} \right] + \left[ {FMN} \right] + K_{d} - \sqrt {\left( {\left[ {APO} \right] + \left[ {FMN} \right] + K_{d} } \right)^{2} - 4\left[ {FMN} \right]\left[ {APO} \right]} }}{2}}}{{\left[ {FMN} \right]}}$$

The fraction in (1) corresponds to the fraction of enzyme-bound FMN at equilibrium $\left( {\frac{{\left[ {HOLO} \right]_{eq} }}{{\left[ {FMN} \right]}}} \right)$.[*HOLO*_*eq*_] was derived from the equation for the dissociation constant of the binding equilibrium,

1a $$K_{d} = \frac{{\left( {\left[ {FMN} \right] - \left[ {HOLO_{eq} } \right]} \right) \times \left( {\left[ {APO} \right] - \left[ {HOLO_{eq} } \right]} \right)}}{{\left[ {HOLO_{eq} } \right]}}$$

as one of the two solutions of the quadratic equation

1b $$\left[ {HOLO_{eq} } \right]^{2} - \left( {K_{d} + \left[ {APO} \right] + \left[ {FMN} \right]} \right) \times \left[ {HOLO_{eq} } \right] + \left[ {FMN} \right] \times \left[ {APO} \right] = 0$$

### Differential scanning fluorimetry (DSF) assays

DSF assays were performed on Real Time PCR Instrument (CFX Connect Real Time PCR system, Bio-Rad). Different experimental conditions were explored to find the optimal protein, FMN, and fluorophore concentrations to be used in DSF experiments. Results indicated that PNPO concentrations lower than 0.5 µM and higher than 5 µM had to be avoided. Moreover, to obtain a good signal of emission fluorescence, no more than 2 µM FMN was added to protein sample. In a typical experiment, 2 μM wild type and variant PNPO forms in 50 mM Na HEPES, pH 7.6, 150 mM NaCl, and Sypro Orange (5× , Thermo Scientific) was mixed with various ligands (total volume of 25 μl) in a 96-well PCR plate. Fluorescence was measured from 25 to 95 °C in 0.4 °C/30 s steps (excitation 450–490 nm; detection 560–580 nm). All samples were run in triplicate. Denaturation profiles were analysed as described in^29^. The stoichiometry of PLP binding to PNPO could not be measured since it required a high concentration of the protein, which was not compatible with the DSF technique.

### Kinetic studies

As reported in^7^, activity assays were carried out in 50 mM Tris–HCl pH 7.6, containing 5 mM 2-mercaptoethanol, using a Hewlett–Packard 8453 diode-array spectrophotometer (Agilent Technologies, Santa Clara, CA), using a 1-cm pathlength cuvette, at 37 °C. The reaction was started by the addition of PNP and kept under constant stirring by a magnetic bar, to ensure a rapid mixing. The progress of the reaction was followed at 414 nm, where the characteristic aldimine product PLP-TRIS absorbs maximally with a molar absorbance coefficient of 4,253 M^−1^ cm^−1^. Kinetic constant measurements were performed using a fixed concentration of either wild type or variant PNPO (2 µM), and varying PNP concentrations from 0.5 to 300 µM. Initial velocities were determined over the first 20 s of the reaction. Because of the low turnover number of the reaction catalyzed by PNPO, we were forced to use a relatively high enzyme concentration with respect to substrate. These conditions are not compatible with classical Michaelis–Menten kinetics, in which the enzyme concentration is much lower than substrate concentration. Nevertheless, the substrate-binding equilibrium can be assumed to be rapidly established because conversion of the substrate into product is relatively slow. Therefore, the values of K_M_ and k_CAT_ were determined from least-squares fitting of initial velocity data as a function of PNP concentration to a quadratic Eq. (2), in which v_i_ is the initial velocity of the reaction, k_CAT_ [E_0_] corresponds to V_MAX_, the maximum velocity of the reaction, [PNP_0_] is the total substrate concentration, [E_0_] is the total enzyme concentration and K_D_ is the dissociation constant of the substrate binding equilibrium $E + PNP \leftrightharpoons E \cdot PNP$ that, assuming a rapid establishment of the equilibrium, is equivalent to K_M_.

2 $$v_{i} = k_{CAT} \left[ {E_{0} } \right]\frac{{\left[ {PNP_{0} } \right] + \left[ {E_{0} } \right] + K_{D} - \sqrt {\left( {\left[ {PNP_{0} } \right] + \left[ {E_{0} } \right] + K_{D} } \right)^{2} - 4\left[ {PNP_{0} } \right]\left[ {E_{0 } } \right]} }}{{2 \left[ {E_{0} } \right]}}$$

Kinetic measurements were also carried out in 50 mM Na-HEPES pH 7.6, containing 5 mM 2-mercaptoethanol. In this case product formation was followed at 388 nm, where PLP absorbs maximally with a molar absorbance coefficient of 5,330 M^−1^ cm^−1^ as calculated from standard PLP solutions, whose concentration was determined in 0.1 N NaOH^7^.

All saturation curves shown in Fig. 4a, obtained by varying PNP at a fixed PLP concentration, were independently analysed using the Michaelis–Menten equation, obtaining apparent V_MAX_ and apparent K_M_ values, which were then used to calculate 1/V_MAX_ and K_M_/V_MAX_. The same data of Fig. 4a were rearranged as initial velocity as a function of PLP concentration measured at different, fixed PNP concentrations (v_X_), obtaining the family of curves shown in Fig. 4c. These curves were globally analysed using Eq. (3), in which all parameters refer to the two-sites mixed modification model shown in Fig. 5b and described in^28^ and v_0_ is the initial velocity obtained at each fixed PNP concentration in the absence of PLP. In the global analysis, all parameters were free to vary and shared, except K_M_ that was fixed at the value determined from the saturation curve obtained in the absence of PLP (1.97 μM) and v_0_, which was fixed at the related value measured at different PNP concentrations in the absence of PLP.

3 $$v_{x} = \frac{{\left( {1 + \beta \frac{{\left[ {PLP} \right]}}{{\alpha K_{I} }}} \right)\left( {1 + \frac{{\left[ {PNP} \right]}}{{K_{M} }}} \right)}}{{1 + \frac{{\left[ {PLP} \right]}}{{K_{I} }} + \frac{{\left[ {PLP} \right]^{2} }}{{\varepsilon K_{I}^{2} }} + \frac{{\left[ {PNP} \right]}}{{K_{M} }}\left( {1 + \frac{{\left[ {PLP} \right]}}{{\alpha K_{I} }}} \right)}}v_{0}$$

### Analysis of PLP binding equilibrium

Analyses of PLP binding to the high affinity site took advantage of FMN fluorescence increase observed upon binding of PLP to PNPO, as described in^7^. Dissociation constants were calculated from saturation curves obtained measuring the protein fluorescence emission intensity as a function of increasing ligand concentration. PLP (from 0.1 to 10 μM) was added to enzyme samples (0.1 μM subunit concentration) at 25 °C in 50 mM Na-HEPES buffer pH 7.6, containing 2-mercaptoethanol, using a 1-cm pathlength quartz cuvette. Fluorescence emission spectra were recorded from 470 to 570 nm upon excitation at 450 nm. Excitation and emission slits were set at 3 nm and 5 nm, respectively. Emission fluorescence values between 520 and 530 nm were averaged and analysed using a quadratic Eq. (4), in which *F*_*rel*_ is the measured relative fluorescence, *F*_*0*_ is fluorescence in the absence of PLP, *F*_*inf*_ is fluorescence at infinite PLP concentration, [*PLP*] is the total PLP concentration, [*PNPO*] stands for the total protein concentration (either wild type or variant forms) and *K*_*D*_ is the dissociation constant of the equilibrium $PLP + PNPO \rightleftharpoons PNPO_{PLP}$, where [*PNPO*_*PLP*_] represents the concentration of PNPO·PLP complex.

4 $$F_{rel} = \left( {F_{\inf } - F_{0} } \right) \times \frac{{\frac{{\left[ {PLP} \right] + \left[ {PNPO} \right] + K_{D} - \sqrt {\left( {\left[ {PLP} \right] + \left[ {PNPO} \right] + K_{D} } \right)^{2} - 4\left[ {PNPO} \right]\left[ {PLP} \right]} }}{2}}}{{\left[ {PNPO} \right]}} + F_{0}$$

The fraction in Eq. (4) corresponds to the fraction of enzyme-bound PLP at equilibrium $\left( {\frac{{\left[ {PNPO_{PLP} } \right]_{eq} }}{{\left[ {PNPO} \right]}}} \right) \cdot \left[ {PNPO_{PLP} } \right]_{eq}$ was derived from the equation for the dissociation constant of the binding equilibrium,

4a $$K_{D} = \frac{{\left( {\left[ {PNPO} \right] - \left[ {PNPO_{PLP} } \right]} \right) \times \left( {\left[ {PLP} \right] - \left[ {PNPO_{PLP} } \right]} \right)}}{{\left[ {PNPO_{PLP} } \right]}}$$

as one of the two solutions of the quadratic equation

4b $$\left[ {PNPO_{PLP} } \right]^{2} - \left( {K_{D} + \left[ {PLP} \right] + \left[ {PNPO} \right]} \right) \times \left[ {PNPO_{PLP} } \right] + \left[ {PNPO} \right] \times \left[ {PLP} \right] = 0$$

Titration experiments carried out to determine the stoichiometry of PLP binding to PNPO were performed using the same method with excitation and emission slits set at 1 and 3 nm, respectively.

Concerning PLP binding to PNPO revealed by DSF measurements, experiments were carried out in 50 mM Na-HEPES buffer pH 7.6, containing 150 mM NaCl, using 2 μM enzyme in the presence of 2 μM FMN and Sypro Orange (5× Thermo Scientific). Melting temperatures, obtained from fitting of fluorescence of data to the Boltzman equation^29^, were analysed as a function of PLP concentration according to the following equation.

5 $$T_{m} = \left( {T_{m}^{\inf } - T_{m}^{0} } \right)\frac{{\left[ {PLP_{0} } \right] + \left[ {E_{0} } \right] + K_{D} - \sqrt {\left( {\left[ {PLP_{0} } \right] + \left[ {E_{0} } \right] + K_{D} } \right)^{2} - 4\left[ {PLP_{0} } \right]\left[ {E_{0 } } \right]} }}{{2 \left[ {E_{0} } \right]}} + T_{m}^{0}$$

In this equation, T_m_ corresponds to the observed melting temperature, T_m_^inf^ is T_m_ at saturating PLP concentration, T_m_^0^ is T_m_ in the absence of PLP, [PLP_0_] is the total PLP concentration and [E_0_] is the total enzyme subunit concentration.

### Data analysis

All data analyses and related figures were performed using the software Prism (GraphPad; https://www.graphpad.com/scientific-software/prism/).

## Supplementary information

Supplementary information
