# Supplementary material for: Molecular characterization of pyridoxine 5′-phosphate oxidase and its pathogenic forms associated with neonatal epileptic encephalopathy
Source: Sci Rep. 2020 Aug 12;10:13621. doi: 10.1038/s41598-020-70598-7 (PMC7424515; doi:10.1038/s41598-020-70598-7)
Supplement: Supplementary file 1 — Supplementary information [file 41598_2020_70598_MOESM1_ESM.pdf]

Supplementary information

Molecular characterization of pyridoxine 5'-phosphate oxidase and its pathogenic forms associated with neonatal epileptic encephalopathy

Anna Barile[1,2], Isabel Nogués[3], Martino L. di Salvo[2], Victoria Bunik[4,5], Roberto Contestabile[2*], Angela Tramonti[1,2*]

Content:

Supplementary Table 1

Supplementary Figure S1

Supplementary Figure S3

Supplementary Figure S4

Supplementary Figure S5

Supplementary Figure S6

Supplementary Figure S7

Supplementary Figure S8

Supplementary Table 1 Summary of pathogenic missense and stop codon variants found in patients affected by PNPOD and effect of PN or PLP administration

PNPO residue	Replacement	Seizure onset	Effect of PN	Effect of PLP	Clinical picture	Reference
Asp33	hz D33V	4 weeks	-	+	mild	[38]
	hz D33V	6 h	-	+	mild	[12]
	hz D33V	4 weeks	Taken during pregnancy -	+	mild	[39]
	hz D33V	3 weeks	+	seizures	mild	[12]
Asp33 Glu120	Heterozygote D33V+ E120K	2 months	+	Not used	mild	[12]
Asp33 Arg116 Arg225	D33V; R116Q; R225C	2 weeks	+	Not used	mild	[12]
Arg95	hzR95H	Not reported	+/-	Not used	death	[17]
	R95C (?)	at birth	Not used	Not used	death	(siblings) [40]
	hzR95C	at birth	-	+	severe	
	hzR95C	15 min	-	Not used	death	(siblings) [40, 41]
	R95C (?)	48 h	-	+/-	severe	
	hzR95C	Few min	-	Not used	death	
	hzR95C	2 h	Not used	+	mild	[12]
Arg116	hzR116Q	5 months	Not used	+	mild	[12]
	hzR116Q	3 h	+	Not used	mild	[12]
	hzR116Q	20 months	+	Not used	mild	[19]
	hzR116Q	3 years	+	Not used	mild	[19]
	hzR116Q	8 months	+	Not used	mild	[19]
Glu50 Arg116	Heterozygous E50K+R116Q	40 days	+	Not used	mild	[42]
Gly118	hzG118R	12 h	+	+	mild	[24]
Arg141	hzR141C+ A94_L97del	6 days	+	Not used	mild	[21]
Arg161	hzR161C	2 days	+	Not used	mild	[43]
Gln174	hzQ174X	at birth	-	+/-	death	[18]
Pro213	hzP213S	90 min	Not used	+	mild	(siblings) [12, 39]
	hzP213S	10 months	Taken during pregnancy	+ Taken as precaution	normal	
His225	hzR225H	I day	+	+ seizures +	severe	[23]
	hzR225H	I day	+	Not used	severe	[21], [12]
	hzR225H	3 days	+	Not used	severe	(siblings) [21]
	hzR225H	I day	+	Not used	mild	
	hzR225H	I day	+	Not used	mild	[21]
	hzR225H	2h	-/+	Not used	death	[44]
	hzR225H	7 days	+	Not used	mild	(siblings) [22]
	hzR225H	2.5 months	+	Not used	mild	
	hzR225H	1 h	+	Not used	mild	[45]
	hzR225C	10 h	-	+	mild	[30]
	hzR225L	Not reported	Not used	+	un	(siblings) [31]
	hzR225L	Not reported	Not used	+	un	
	hzR225L	Not reported	Not used	+	un	[31]
	hzR225L	Not reported	Not used	+	un	[31]
Arg225 Arg116	hzR225H+R116Q	30 min	+	Seizures	severe	[12]
	R225H+R116Q (?)	25 min	-	Not used	death	[12] [21]
	hz R225H+R116Q	10 h	+	Seizures	Severe	
	R225H+R116Q (?)	Not reported	-	Not used	death	[21]
	hz R225H+R116Q	3h	-/+	Seizures	severe	
	hz R225H+R116Q	2 day	+	Not used	mild	
	hz R225H+R116Q	3 h	+	Not used	mild	[21]
Arg229	hzR229W (?)	at birth	Not used	Not used	death	[13]
	hzR229W (?)	at birth	Not used	Not used	death	(siblings) [13]
	hzR229W ?	at birth	+/-	Not used	death	(siblings) [13]
	hzR229W ?	at birth	+/-	Not used	death	
	hzR229Q	3 h	-	+	mild	[31]
	hzR229Q	1 day	Not used	+	mild	[23]
Stop	hz X262Q	30 min	-	Not used	death	(siblings) [13]
	hz X262Q	30 min	-	Not used	death	

In the "replacement" column, hz indicates homozygous. The question mark indicates that the replacement was inferred from family history. In the "effect" columns, + indicates that the administration of treatment lead to a decrease in seizure frequency or a cessation of seizures, whereas – indicates that the treatment has no effect, resulting in a recurrence of seizures.

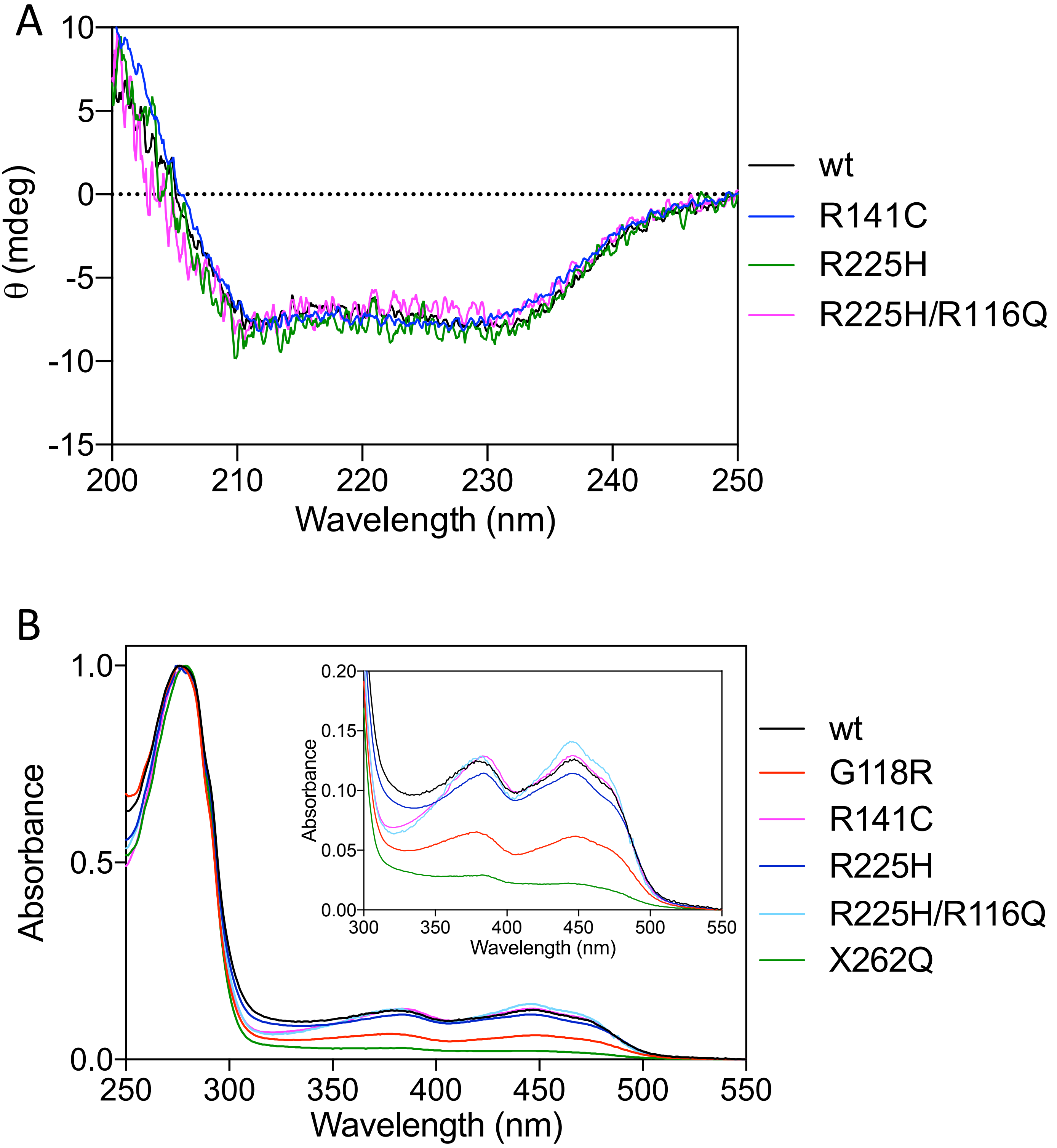

Supplementary Figure S1: Far-UV CD (A) and absorption (B) spectra of the indicated PNPO variants. Inset in panel B is an expanded view of the visible region of the same absorption spectra, showing absorption bands due to FMN. For each protein sample three independent preparations were analysed, although in the figure wild type, G118R, R141C, R225H, R116Q/R225H, and X262Q, with FMN saturation of 71%, 30%, 74%, 64%, 84% and 10%, respectively, were represented. All spectra were measured in 20 mM potassium-phosphate buffer, pH 7.6. Images were generated using the software Prism 8 (GraphPad; https://www.graphpad.com/scientific-software/prism/).

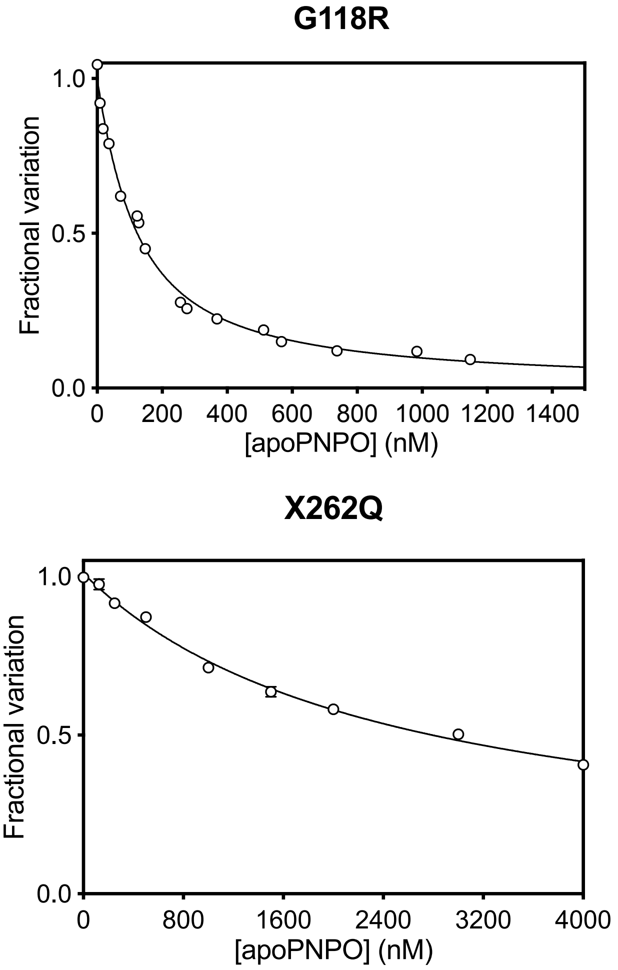

Supplementary Figure S2: FMN binding to selected apo-PNPO variants. The figure shows the saturation curves of FMN binding obtained with the PNPO variants that showed the highest K_D values. Data were analysed as explained in the Methods section. Images were generated using the software Prism 8 (GraphPad; https://www.graphpad.com/scientific-software/prism/).

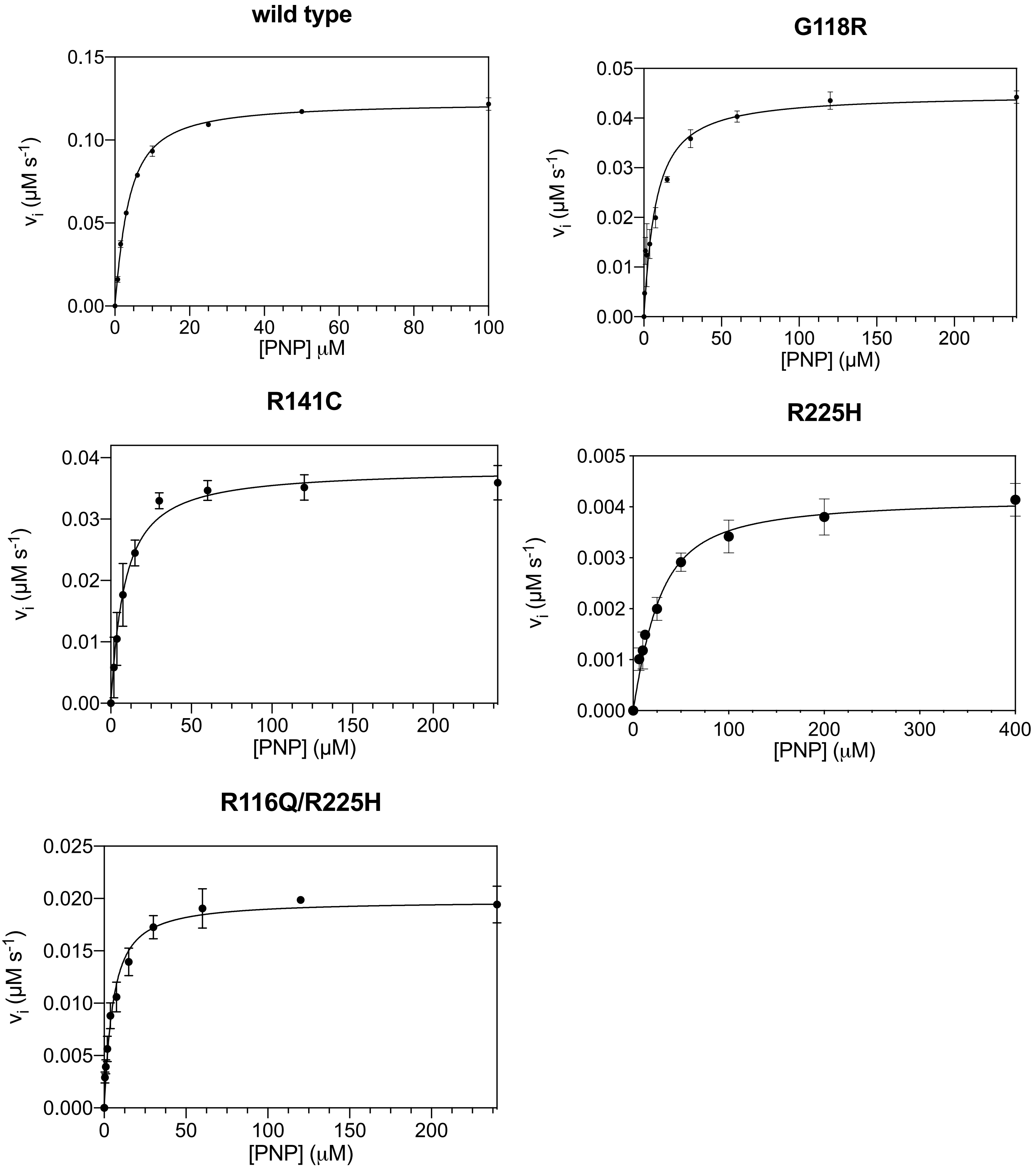

Supplementary Figure S3: Saturation curves obtained with PNPO variants in TRIS buffer. Initial velocity of the reaction catalysed by the wild type and the indicated PNPO variants (2 μM) as a function of substrate concentration. These saturation curves were analysed using the quadratic equation (2), obtaining the kinetic parameters listed in the Table 1. Images were generated using the software Prism 8 (GraphPad; https://www.graphpad.com/scientific-software/prism/).

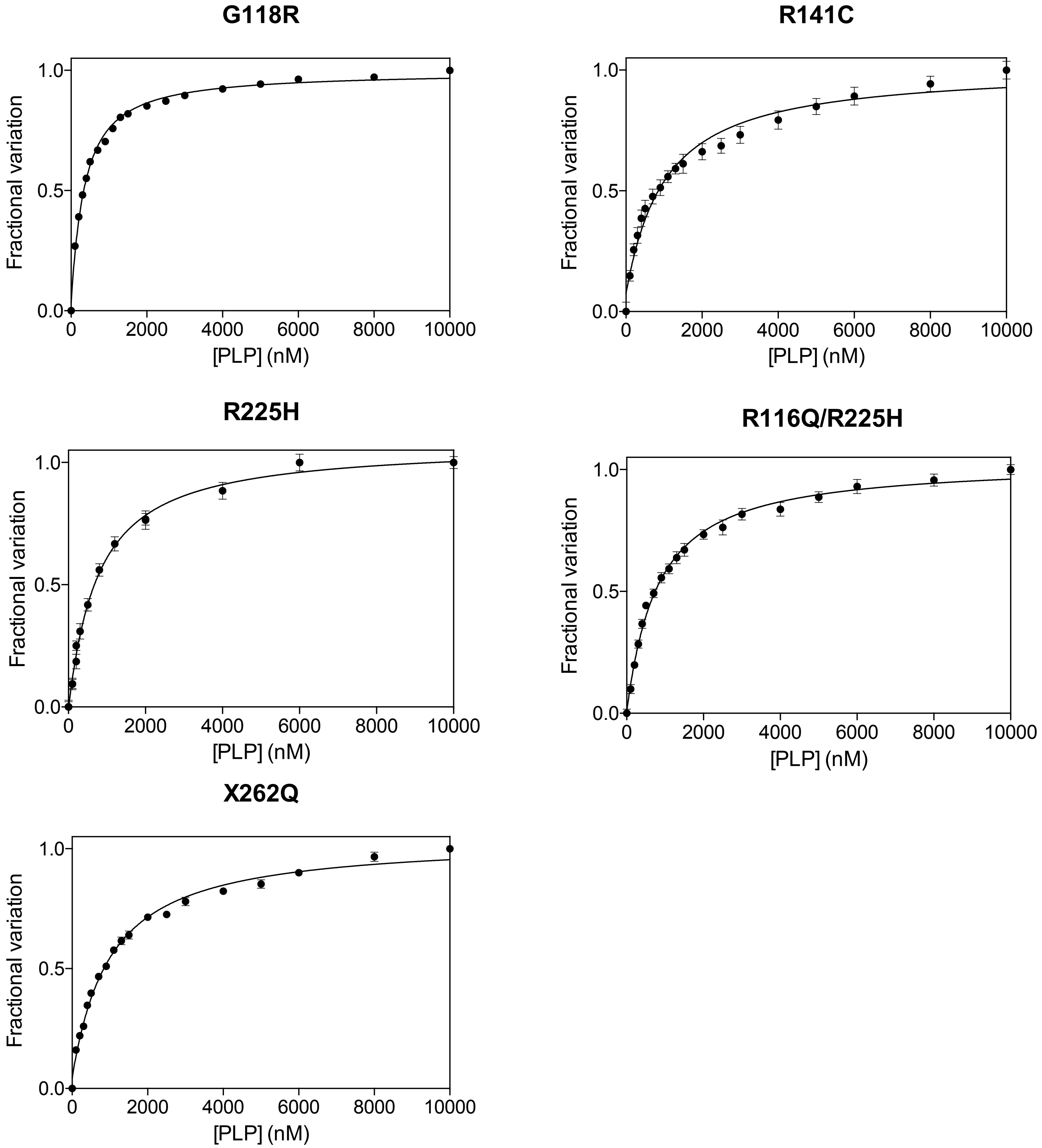

Supplementary Figure S4: Analysis of PLP binding equilibrium of PNPO variants. PLP binding curves obtained with 100 nM PNPO variants. Reported data are the average ± standard deviation of three independent measurements. Data were fitted to equation (3) and the resulting K_I values were reported in Table 1. Images were generated using the software Prism 8 (GraphPad; https://www.graphpad.com/scientific-software/prism/).

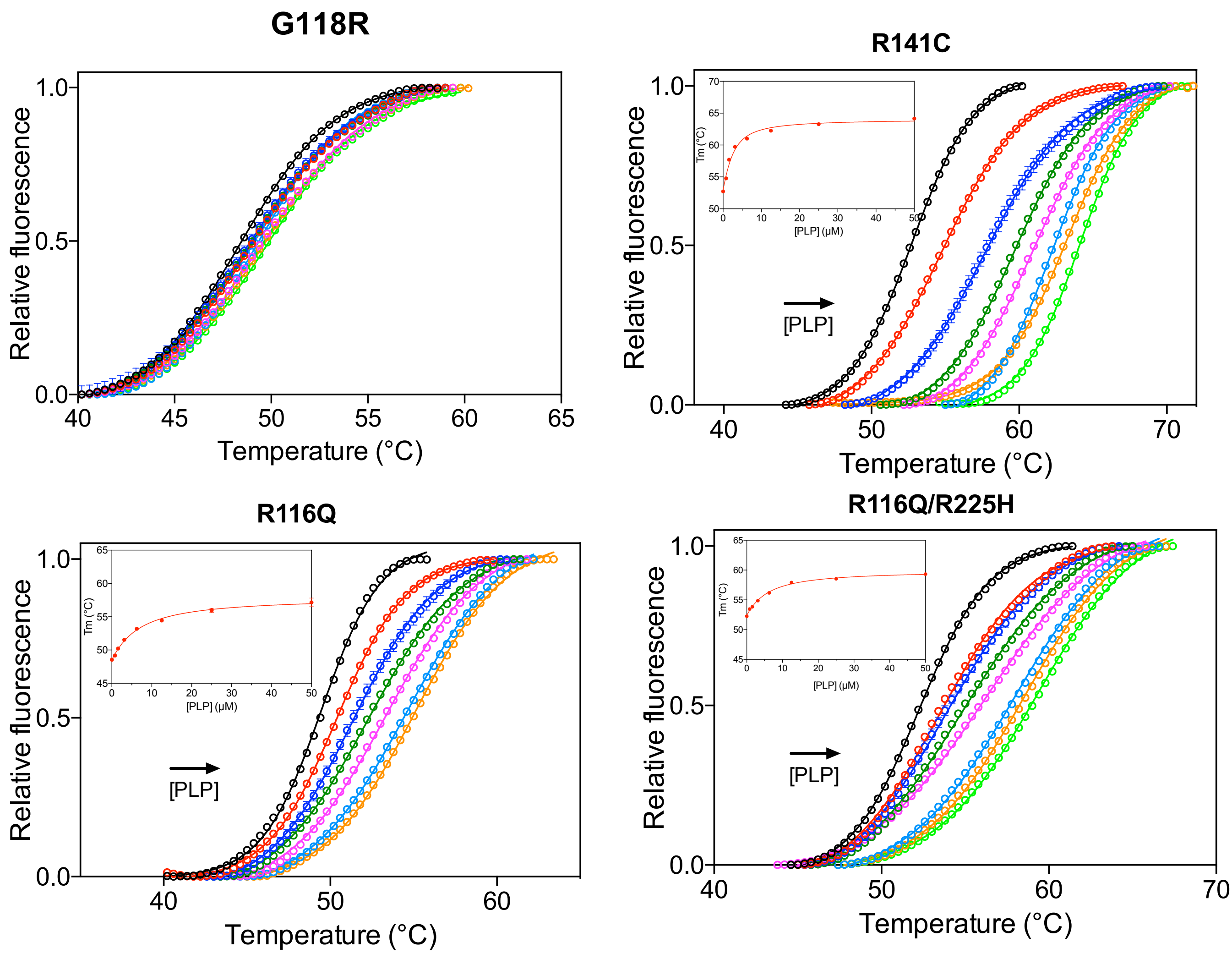

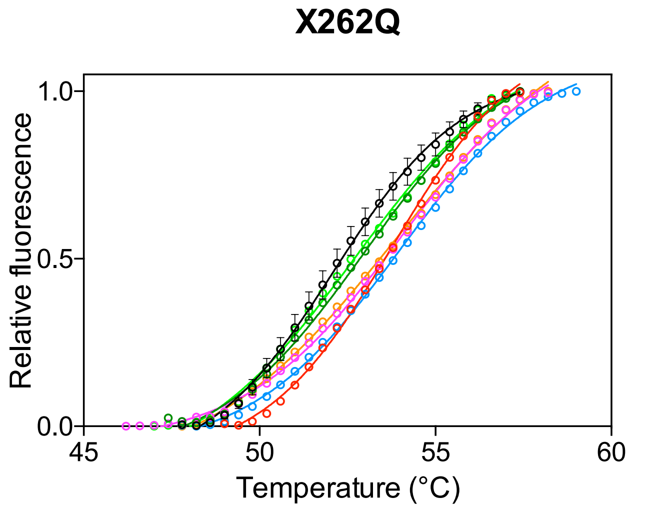

Supplementary Figure S5: DSF measurements with PNPO variants in the presence of different PLP concentrations. Fluorescence change is expressed as fractional variation as a function of temperature. The experiment was carried out using 2 µM enzyme and different PLP concentrations (0, 0.78, 1.56, 3.13, 6.25, 12.5, 25 and 50 µM). Thermal denaturation data were fitted to the Boltzmann equation to obtain melting temperatures. Each curve is the average of three independent experiments. In the insets, the saturation curves obtained by plotting the melting temperatures as a function of the PLP concentrations. Data were analysed using the quadratic equation (4) to estimate the dissociation constant reported in Table 1. Images were generated using the software Prism 8 (GraphPad; https://www.graphpad.com/scientific-software/prism/).

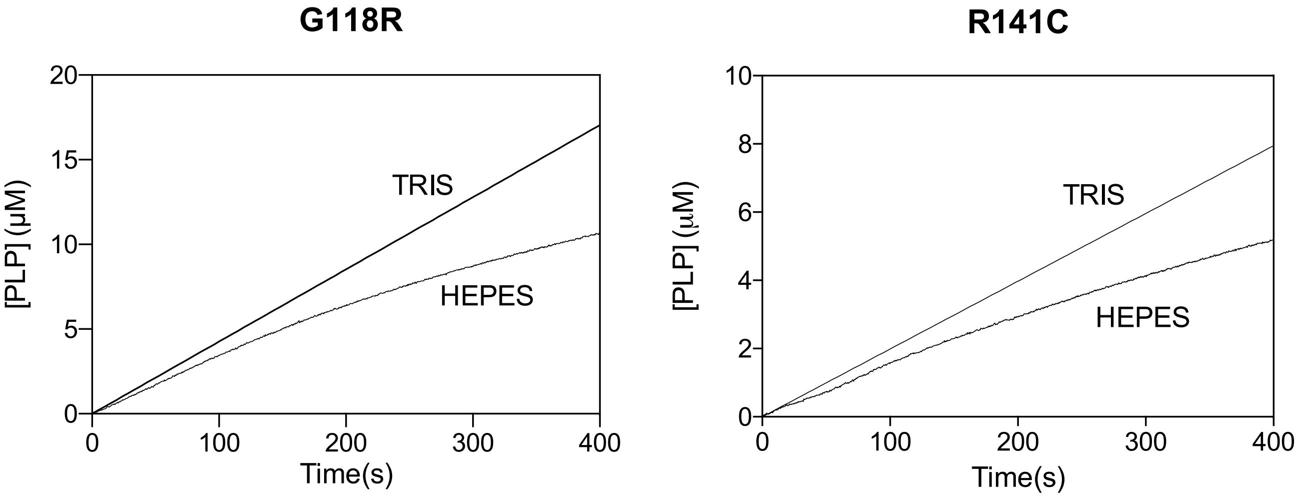

Supplementary Figure S6: Kinetics of PNP oxidation to PLP in TRIS and HEPES buffers Comparison of kinetics carried out in 50 mM TRIS-HCl and 50 mM Na-HEPES buffers at pH 7.6, obtained using 1 µM PNPO variants and 100 µM PNP. Images were generated using the software Prism 8 (GraphPad; https://www.graphpad.com/scientific-software/prism/).

```
                                                                   D33             E50
H. sapiens        -6 aa --GVTATFGRPAEWPGYLS-HLCGR---SAAMDLGPMRKSYRGDREA-FEETHL  53
S. scrofa         -6 aa --GVIVTFGRPAQWPRYAR-HLCSR---GAVMDLGPMRKSYCGEREA-FEETQL  53
B. taurus         -6 aa --SVIVTFGRPAEWPRCLR-HLCSR---GAAMDLGPMRKTYRGDPEA-FEETHL  53
O. orca           -6 aa --GVIVTFGRPAEWPRCLR-HLCSR---GAVMDLGPMRKSYRGDREA-FEETQL  53
C. lupus          -6 aa --GVTVTFGRPAGWPRYLH-HLCGG---GVVMDLGPMRKSYRGDREA-FEETQL  53
R. norvegicus     -6 aa --SVTVTFRRPAKWPGYFR-HLCCR---GAVMDLGPMRKSYRGDREA-FEEAHL  53
C. elegans        --------------------------------METPSIDIQNIRAKYLNSHDPYLLESKL  28
M. domestica      --------------------------------MSAANVPLSALRLKYMEKKQA-FLEENI  27
S. cerevisiae     --------------------------MTKQ-AEETQKPIIFAPETYQYD--KFTLNEKQ  30
Synechocystis     ----------------------------------MDGVSLADLRLNYTQGG---LIEAE-  22
C. freundii       ------------------------------------MQPDIAGIRTNYSRQS---LSEAD-  21
E. coli           -----------------------------MSDNDELQQIAHLRREYTKGG---LRRRD-  26
P. somniferum     302 aa -PPTIAEKYQLQLPPYIGTSMCVRIGKPPKVDISALRENYVSPA---FLEDQ-  350
Q. suber          300 aa -PPSVADKYKLRLPPYPGTSMCVRIGKAPKIDISALRENYISPE---LLEEQ-  348
A. thaliana       221 aa -PPSVAEKYKLELPSYPGTSMCVRIGKPPKVDISAMRVNYVSPE---LLEEQ-  269
                                                          :        *       . .

                                                R95
H. sapiens        TSLDPVKQFAAWFEEAVQCPD--IGEANAMCLATCTRDGKPSARMLLLKGFGKDGFRFFT  111
S. scrofa         TSLDPVKQFAAWFEEAVQCPA--IGEANAMCLATCTRDGRPSARMVLLKGFGKDGFRFFT  111
B. taurus         TSLDPVKQFAAWFEEAVQCPD--IMEANAMCLATCTRDGKPSARMVLLKGFGKDGFRFFT  111
O. orca           TSLNPMKQFAAWFEEAVQCPD--IGEANAMCLATCTRDGKPSARMVLLKGFGKDGFRFFT  111
C. lupus          TSLDPMKQFATWFEEAVQCPD--IGEANAMCLATCTRDGKPSARMVLLKGFGKDGFRFFT  111
R. norvegicus     TSLDPMKQFASWFEEAVQCPD--IGEANAMCLATCTRDGKPSARMLLLKGFGKDGFRFFT  111
C. elegans        PTTSPFELFDIWFRNVASQSDLTFEEINAVSLSTVGKDLRPSSRMVLLKAYTPTGFSFYT  88
M. domestica      RVKEPITLFKEWMNQALQTEE--IIEPNAASLATVGSDGCPSNRFVLLKDISDEGFTFFT  85
S. cerevisiae     LTDDPIDLFTKWFNEAKEDPRETLPEAITFSS-AELPSGRVSSRILFKELDHRGFTIYS  89
Synechocystis     VADHPFAQFHIWLQQAIA-AE--LPEPNAMTLSTLSEEGHPVGRMVLLKGLDERGFVFYT  79
C. freundii       IEANAIKQFAKWWQEAVD-SK--IDEVNAMTLATASPDGIPSARIVLLKGFSEDGFMFFT  78
E. coli           LPADPLTLFERWLSQACE-AK--LADPTAMVVATVDEHGQPYQRIVLLKHYDEKGMVFYT  83
P. somniferum     LEADPIDQFRKWFDDALV-AG--LHEPNAMALSTSNKNGKPSSRMVLLKGVDKEGFVWYT  407
Q. suber          VEADPIDQFCKWFDDAVA-AS--LKEPNAMALSTAGKDGKPSSRMVLLKGVDKDGFVWYT  405
A. thaliana       VETDPTVQFRKWFDEAVA-AG--LRETNAMALSTANKDKKPSSRMVLLKGFDENGFVWFT  326
                            *  *  :.      : :  :    :           *::*:*    *:  ::

                    R116 G118                 R138 R141               R161
H. sapiens        NFE-SRKGKELDSNPFASLVFYWEPLNRQVRVEGPVKKLPEEEAECYFHSRPKSSQIGAV  170
S. scrofa         NFQ-SRKGKELDSNPFASLVFYWEPLNRQVRLEGPVKKLPEEEAESYFHSRPRSSQIGAV  170
B. taurus         NFE-SRKGKELDSNPFASLVFYWEPLHRQVRVEGPVKKLPEEEAECYFHSRPKSSQIGAV  170
O. orca           NFE-SRKGQELDSNPFASLVFYWEPLNRQVRVEGPVKKLPEEEAECYFHSRPKSSQIGAV  170
C. lupus          NFE-SRKGRELDSNPFASLVFYWEPLNRQVRVEGSVKKLPEEEAECYFHSRPKSSQIGAV  170
R. norvegicus     NYE-SRKGKELDSNPFASLVFYWEPLNRQVRVEGPVKKLPEKEAENYFHSRPKSSQIGAV  170
C. elegans        NYT-SRKGNQLEENPNAAMLFYWPKVNRQIRVEGVVEKLPDEMAVAYWNSRPVASRIGSK  147
M. domestica      NYG-SRKAQNIASNPNVAMTLYWCPLRRSIRIEGVAEKIPQEDSLKYFHERPRASQIGAL  144
S. cerevisiae     NWGTSRKAHDIATNPNAAIVFFWKDLQRQVRVEGITEHVNRETSERYFKTRPRGSKIGAW  149
Synechocystis     NYD-SAKGQQLTAHPWAGLVFWWAALERQVRVDGQVEKIDPAESDAYFQSRPRGSQLGAW  138
C. freundii       NYN-SYKGKQLAENPKACLVFFWKELERQVRITGIIKKVSAAESDEYFYSRPIGSQIGAT  137
E. coli           NLG-SRKAHQIENNPRVSLLFPWHTLERQVMVIGKAKRLSTLEVMKYFHSRPRDSQIGAW  142
P. somniferum     NYE-SQKGHDLSDNPRASLLFYWDGLNRQVRVEGPVQKVPEEESENYFHSRPRGSQIGAI  466
Q. suber          NYD-SRKAHDLSDNPHASLLFYWDGLNRQVRVEGSVQRVSDEESEQYFHSRPRGSQIGAI  464
A. thaliana       NYE-SKKGSDLSENPSAALLFYWEILNRQVRIEGPVERIPESESENYFHSRPRGSQIGAI  385
                  *   * *. ::  :* . : : *  :.*.: : *  :::       *:  **  *::*:

                                                             P213        R225
H. sapiens        VSHQSSVIPDREYLRKKNEELEQLYQ--DQ-EVPKPKSWGGYVLYPQVMEFWQGQTNRLH  227
S. scrofa         VSHQSSVIPDREYLRKKNEELEQLYQ--EQ-EVPRPKYWGGYILYPQVIEFWQGQTNRLH  227
B. taurus         VSHQSSVIPDREYLRKKNKELEQLYQ--EQ-EVPKPKYWGGYILYPQVMEFWQGQTNRLH  227
O. orca           VSHQSSVIPDREYLRKKNEELEQLYQ--EK-EVPKPKYWGGYTLYPQVMEFWQGQTNRLH  227
C. lupus          VSHQSSVIPNREYLRKKNEELEQLYQ--EQ-EVPKPKYWGGYILYPQVMEFWQGQTNRLH  227
R. norvegicus     VSRQSSVIPDREYLRKKNEELGQLYR--EQ-EVPKPEYWGGYILYPQVMEFWQGQTNRLH  227
C. elegans        SSDQSKVVPDREFLESKKVALTELSVREGAQAITKPESWGGYHLIPRYFEFWQGQSDRLH  207
M. domestica      ASEQSSRIPSRKHLDEIEKKIKQELG--DDKAVPL-PNWGGYLIRPKLIEFWQGQTDRLH  201
S. cerevisiae     ASRQSDVIKNREELDELTQKNTERFK--DAEDIPCPDYWGGLRIVPLEIEFWQGRPSRLH  207
Synechocystis     ASPQSRIVGDRQELEDNLARWEKQYE--NQ-SIPRPPHWGGFRVIPHRIEFWQGRPSRLH  195
C. freundii       VSPQSSIIESREWLEEKYAAKEKESA--TG-KILRPANWGGYILTPVLIEFWQGRPSRLH  194
E. coli           VSKQSSRISARGILESKFLELKQKFQ--QG-EVPLPSFWGGFRVSLEQIEFWQGGEHRLH  199
P. somniferum     VSKQSSIVPGREVLYQEYEDLENRFS--DGTIIPKPKHWGGYRLKPELVEFWQGQPSRLH  524
Q. suber          VSKQSTVVPGRHVLLQQYKELEEKFS--DGSLIPKPKHWGGYRLKPELFEFWQGQKSRLH  522
A. thaliana       VSKQSSVVPGRHVLYDEYEELTKQYS--DGSVIPKPKNWGGFRLKPNLFEFWQGQPSRLH  443
                   * **  :  *  *  .         :          :    ***   :   .*****   ***

                   R229
H. sapiens        DRIVFRRGLPTGDSPLGPMTHRGEEDWLYERLAP      261
S. scrofa         DRIIFRRGPPTGDSPLGPMTHRGEEDWLYERLAP      261
B. taurus         DRIVFRRGLLTGDSPLGPMTHRGEEDWVYERLAP      261
O. orca           DRIAFRRGLPTGDSPLGPMTHRGEEDWLYERLAP      261
C. lupus          DRIIFRRGLPTGDSPLGPMTHRGEEDWLYERLAP      261
R. norvegicus     DRIVFRRGLATGDSPLGPMTHHGEEDWVYERLAP      261
C. elegans        DRIVFER----------------DVDVWLLKRLSP      226
M. domestica      DRIVFRKYSGVEKEIDNVLVHPAENGWVYERLAP      235
S. cerevisiae     DRFVYRR-------------KTENDPWKVVRLAP      228
Synechocystis     DRLQFNL-----------LD----GQWHRQRLAP      214
C. freundii       DRFEYSL-----------QE---NGSWKAERLAP      214
E. coli           DRFLYQR----------------ENDAWKIDRLAP      218
P. somniferum     DRLQYSL-----------REIDGKWAWKVDRLAP      547
Q. suber          DRLQYSP-----------HEINGQQVWKIERLAP      545
A. thaliana       DRLQYSL-----------QDVNGNPAWKIHRLAP      466
                  **: :                        *   **:*
```

Supplementary Figure S7: Multiple alignment of PNPO sequences carried out with Clustal omega. Mutated residues are indicated

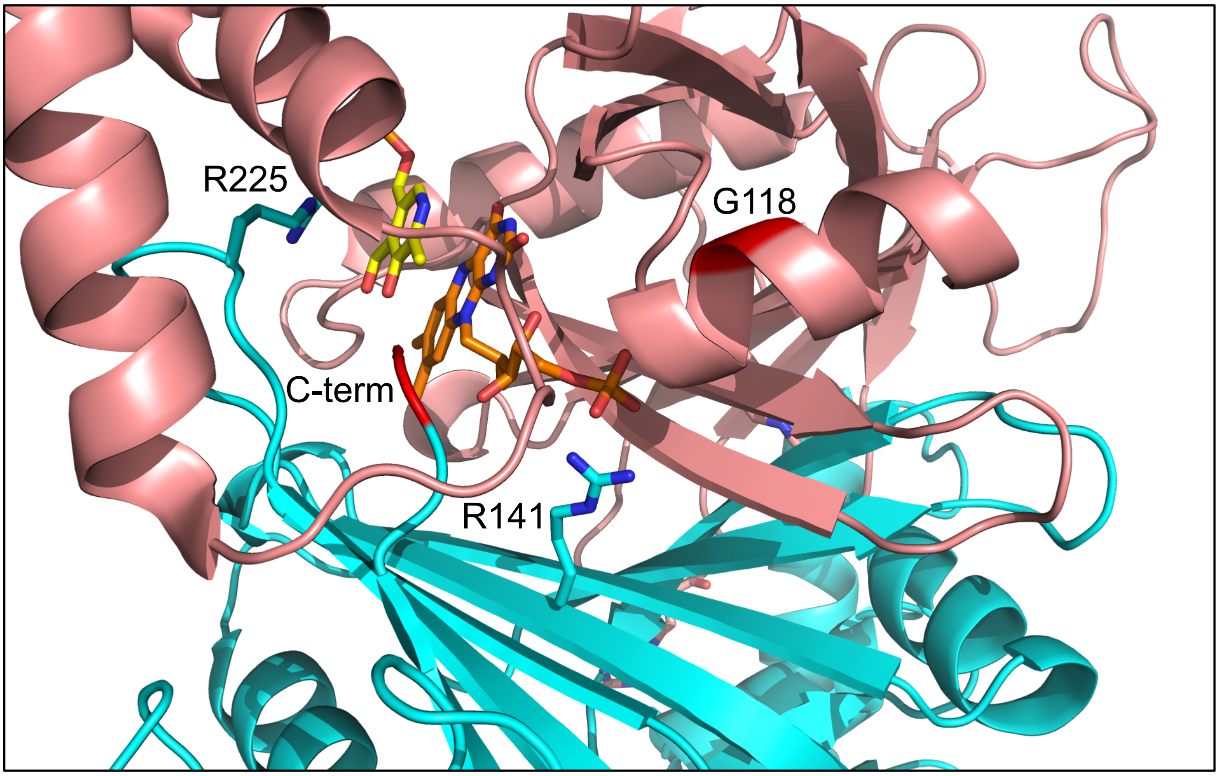

Supplementary Figure S8: Close-up view of the active site of human PNPO (PDB: 1NRG), showing the mutated residues studied in our investigation as sticks. The two subunits of the human PNPO dimer are shown in cyan and magenta colour, respectively. FMN and PLP in the active site are shown as sticks, in orange and yellow colour, respectively. The R141 residue interacts with the phosphate group of FMN. The G118 residue, which is a part of the α2-helix, is shown in red. The N-terminal part of this helix electrostatically interacts with the phosphate group of FMN. Moreover, the position of the C-terminal end of the protein, pointing towards the active site, is shown. The image was created using PyMOL v1.7.4.5 Edu Enhanced (https://pymol.org/2/).
